# Free‐breathing phase‐sensitive inversion recovery T_1_ ‐weighted imaging for improved visualization of focal liver lesions

**DOI:** 10.1002/mrm.70042

**Published:** 2025-09-08

**Authors:** Yavuz Muslu, Julius F. Heidenreich, Jan‐Peter Grunz, Ty A. Cashen, Sagar Mandava, Ali Pirasteh, Diego Hernando, Scott B. Reeder

**Affiliations:** ^1^ Department of Biomedical Engineering University of Wisconsin‐Madison Madison Wisconsin USA; ^2^ Department of Radiology University of Wisconsin‐Madison Madison Wisconsin USA; ^3^ Department of Diagnostic and Interventional Radiology University Hospital Würzburg Würzburg Germany; ^4^ GE Healthcare Waukesha Wisconsin USA; ^5^ Department of Medical Physics University of Wisconsin‐Madison Madison Wisconsin USA; ^6^ Department of Electrical and Computer Engineering University of Wisconsin‐Madison Madison Wisconsin USA; ^7^ Department of Medicine University of Wisconsin‐Madison Madison Wisconsin USA; ^8^ Department of Emergency Medicine University of Wisconsin‐Madison Madison Wisconsin USA

**Keywords:** free‐breathing, gadoxetic acid, hepatobiliary phase T_1_‐weighted, inversion recovery, lesion characterization, liver–lesion contrast

## Abstract

**Purpose:**

Gadoxetic acid‐enhanced hepatobiliary phase T_1_‐weighted (T_1_w) MRI is effective for the detection of focal liver lesions but lacks sufficient T_1_ contrast to distinguish benign from malignant lesions. Although the addition of T_2_, diffusion, and dynamic contrast‐enhanced T_1_w imaging improves lesion characterization, these methods often do not provide adequate spatial resolution to identify subcentimeter lesions. This work proposes a high‐resolution, volumetric, free‐breathing liver MRI method that produces colocalized fat‐suppressed, variable T_1_w images from a single acquisition, thereby improving both lesion detection and characterization.

**Theory and Methods:**

This method combines stack‐of‐stars radial sampling, magnetization preparation, and chemical shift encoding to enable free‐breathing, T_1_w imaging with water/fat separation. A model‐based image reconstruction algorithm reconstructs images from highly undersampled k‐space data. Pseudo‐T_1_ relaxation maps are calculated from the variable T_1_w images. The feasibility of this method was investigated in patients undergoing clinical contrast‐enhanced MRI examinations for detection and characterization of focal liver lesions at both 1.5 and 3.0 T. An expert reader study was conducted to evaluate the method's performance, compared with the hepatobiliary phase‐navigated T_1_w MRI based on image quality and lesion conspicuity.

**Results:**

Expert readers found that at shorter inversion times (TIs) (˜500 ms), the proposed method had superior liver–lesion contrast for characterizing simple cysts and metastases, compared with navigated T_1_w images.

**Conclusion:**

The proposed method produces colocalized fat‐suppressed, variable T_1_w images from a single acquisition that may improve focal liver lesion detection and characterization.

## 1 INTRODUCTION

The liver receives dual blood supplies from the hepatic artery and portal vein, providing direct pathways for malignant cells from different organs in the gastrointestinal tract to metastasize to the liver. As a result, liver metastases are highly common among many cancer patients, in whom approximately 40% of metastatic cases include liver metastasis.^1^ Colorectal cancer is one of the most likely cancer types that result in liver metastases. Various studies show that 30%–50% of colorectal cancer patients develop liver metastases over the course of their disease.^2^, ^3^, ^4^, ^5^ If untreated, patients with colorectal liver metastases have median survival time of 5–20 months, with 5‐year survival rate of 90% for stage I–II, 71% for stage III, and 14% for stage IV disease,^6^, ^7^, ^8^, ^9^, ^10^, ^11^ whereas overall median survival time of liver metastases is 4 months.^1^ Curative partial hepatectomy is the only effective treatment in patients with liver metastases proven to offer long‐term survival.^7^, ^8^, ^9^, ^12^

Preoperative imaging is vital for overall staging and the assessment of surgical resectability of metastases by determining the number and lobar distribution of lesions. For these reasons, imaging has an indirect but important impact on survival after curative hepatectomy.^13^, ^14^ Historically, a minimum negative surgical margin of 1 cm was considered necessary to minimize recurrence and improve 5‐year survival rates.^13^, ^15^ This requirement limited resectability because such surgical margins are not always achievable if multiple lesions are present or lesions are close to critical anatomical structures.^16^ More recent studies suggest that as long as metastatic tissue is completely removed, the margin size does not affect survival rates, expanding resectability criteria and increasing treatment availability to more patients.^15^, ^17^ Consequently, accurate and high‐resolution imaging of liver metastases is increasingly important.

Contrast‐enhanced T_1_‐weighted (T_1_w) MRI has become an essential tool for detecting liver metastases, due to higher sensitivity for small (<1 cm) focal liver lesions (FLL) compared to other imaging methods.^18^, ^19^, ^20^ Among gadolinium‐based contrast agents, gadoxetic acid (GA) is the most widely used hepatobiliary agent due to its combined extracellular and hepatocyte‐specific contrast properties, enhancing lesion detection. Lesions of non‐hepatocyte origin such as metastases, as well as benign lesions such as cysts and cavernous hemangiomas, appear as dark voids on delayed hepatobiliary phase (HBP) T_1_w imaging, typically performed 15–25 min after the GA infusion. Thus, GA‐enhanced HBP T_1_w imaging is exquisitely sensitive for the detection of FLLs but may lack specificity to differentiate metastases from benign lesions.

For this reason, characterization of FLLs currently requires the addition of other sequences including dynamic contrast‐enhanced (DCE)‐T_1_w, in‐ and opposed‐phase (IOP) T_1_w imaging, T_2_‐weighted (T_2_w) imaging, and/or DWI. However, GA‐enhanced HBP T_1_w methods, especially those using free‐breathing and optimized flip angles for ultrahigh resolution and optimized lesion–liver contrast, often outperform other methods.^21^ Consequently, it is a common clinical conundrum that a small lesion of non‐hepatocyte origin seen on GA‐enhanced HBP T_1_w imaging is occult on every other sequence. Indeed, several studies have reported on the challenges of detecting small liver lesions with T_2_w and DWI methods. Gatti et al. found that detection sensitivity for subcentimeter lesions was 85% for DWI and ranged between 27% and 44% for T_2_w MRI.^22^ Such lesions are characterized as *indeterminate*, creating uncertainty in both staging and treatment planning.^21^, ^22^, ^23^, ^24^, ^25^, ^26^

Additionally, conventional GA‐enhanced HBP T_1_w methods utilizing Cartesian sampling strategies are susceptible to motion artifacts that manifest as ghosting. These methods often employ navigated or respiratory‐triggered image acquisitions, thereby reducing imaging efficiency.

In this study, we develop a novel free‐breathing, high‐resolution imaging method with enhanced, variable T_1_ contrast as an alternative to current standard‐of‐care GA‐enhanced hepatobiliary phase navigated T_1_w MRI for improved detection and characterization of small liver lesions. We validate and demonstrate the feasibility of this volumetric, inversion recovery (IR)‐prepared T_1_w imaging approach, which incorporates radial sampling and robust fat suppression.

## 2 THEORY

### 2.1 T_1_ contrast of focal liver lesions

Conventional GA‐enhanced HBP T_1_w MRI methods utilize T_1_w spoiled gradient echo (SGRE) imaging. Due to the lack of a strong T_1_ contrast, SGRE‐based T_1_w MRI methods cannot delineate the differences between benign and malignant lesions.^27^ IR is a well‐established method to increase the dynamic range of T_1_ contrast, thereby improving the tissue discrimination based on T_1_ relaxation.^28^, ^29^

**FIGURE 1:**
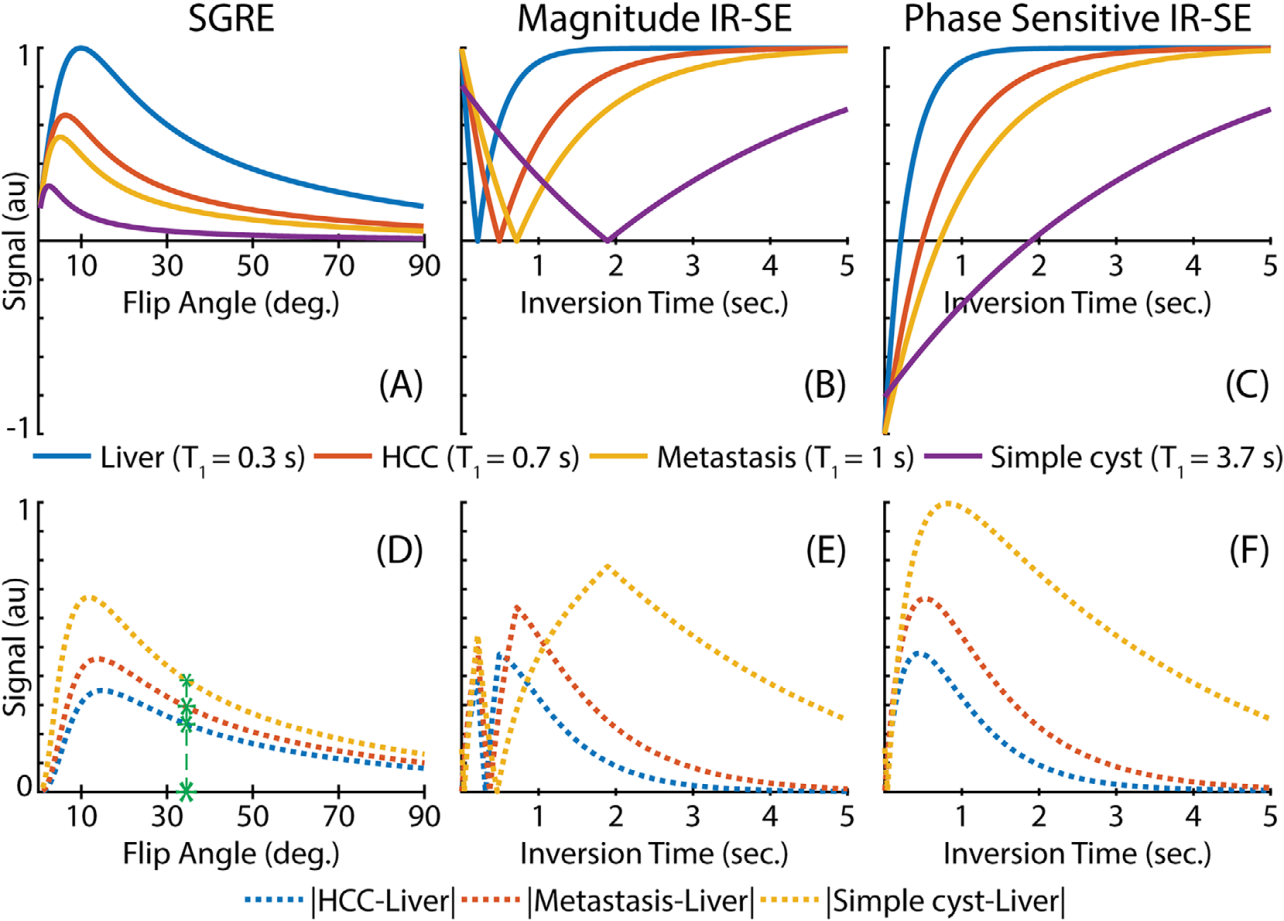
Phase‐sensitive inversion recovery methods improve T_1_ contrast difference between the liver, benign and malignant lesions. (A–C) Normalized T_1_ contrasts for SGRE, magnitude IR‐SE, and phase‐sensitive IR‐SE methods. (D–F) Absolute T_1_ contrast differences between post‐contrast liver (T_1_ ˜ 300 ms) and liver lesions (hepatocellular carcinoma (T_1_ ˜ 700 ms), metastasis (T_1_ ˜ 1000 ms), and simple cyst (T_1_ ˜ 3700 ms). Simulations confirm that phase‐sensitive IR‐SE method can achieve larger T_1_ contrast differences compared to SGRE and is less sensitive to choice of TI compared to magnitude IR‐SE. (D) Green stars illustrate FAs used in clinical GA‐enhanced imaging protocols and corresponding T_1_ contrast differences between liver and lesions. Relevant simulation parameters are as follows: TR (SGRE) = 5 ms, TR (IR‐SE) = 6 s, FA (IR‐SE) = 90°. FA, flip angle; GA, gadoxetic acid; IR‐SE, inversion recovery spin echo; SGRE, spoiled gradient echo.

Figure 1A–C illustrates simulated signal intensities of post‐contrast liver, hepatocellular carcinoma, metastasis, and simple cysts using SGRE, magnitude IR‐spin echo (SE), and phase‐sensitive (PS) IR‐SE methods across various flip angles and inversion times.^30^ Figure 1D–F demonstrates expected absolute signal differences between post‐contrast liver and benign and malignant lesions obtained with the same methods. Magnitude and phase‐sensitive IR‐SE methods are able to achieve larger T_1_ contrast differences compared to SGRE. However, with magnitude reconstruction, the relative liver–lesion T_1_ contrast depends heavily on the choice of TI. In fact, T_1_ contrast differences between benign and malignant lesions may decrease as a result of a suboptimal TI choice. PS reconstruction, however, preserves the full dynamic range of T_1_ contrast.

### 2.2 Inversion recovery, chemical shift‐encoded, stack‐of‐stars acquisition

The proposed method builds upon a method proposed by Kellman et al., where an electrocardiogram‐synchronized single‐slice IR image and its phase reference are acquired during a breath‐hold for delayed myocardial enhanced T_1_w cardiac MRI.^28^ In the current work, we extend this approach to utilize a SGRE radial stack‐of‐stars (SoS) k‐space trajectory for volumetric, free‐breathing, phase‐sensitive inversion recovery (PSIR) imaging.^31^, ^32^, ^33^, ^34^ The proposed method is more robust to motion artifacts than Cartesian trajectories due to radial sampling in the *k*_x_‐*k*_y_ plane. Furthermore, incoherent aliasing artifacts created by radial sampling are well suited for compressed sensing reconstruction and retrospective motion compensation.^32^, ^33^, ^34^, ^35^, ^36^

**FIGURE 2:**
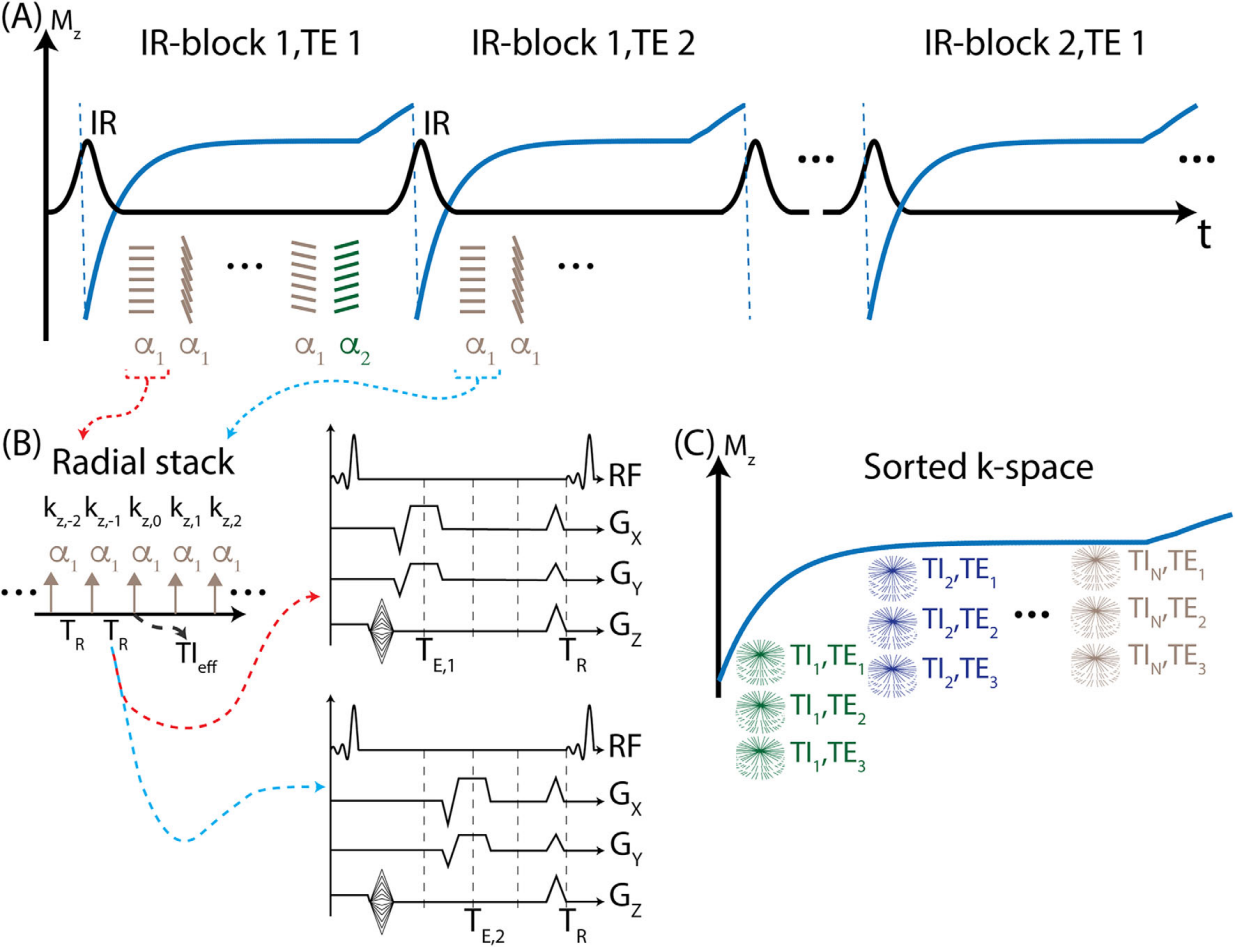
The proposed data acquisition strategy provides volumetric images with T_1_ and chemical shift contrast. (A) Each IR block consists of an adiabatic inversion pulse, radial stacks of k_z_‐readouts, and an idle period. IR blocks are repeated for each TE to obtain chemical shift contrast. A small FA is used to acquire the last TI_eff_ for PS reconstruction. $\left(a_{1},a_{2}\right)$ represents the FA pair. (B) TI_eff_ is defined as the acquisition time of the center of the k‐space for each radial stack. (C) The proposed image acquisition strategy delivers multi‐contrast images acquired at different TI_eff_ and TE points. Note that gradient waveforms are only illustrative. *M*_*z*_, longitudinal magnetization; PS, phase‐sensitive; TI_eff_, effective TI.

A schematic of the proposed image acquisition is outlined in Figure 2. Following an adiabatic nonselective IR pulse, a series of radial stacks of gradient echo *k*_z_‐readouts is acquired. The number of radial stacks is chosen based on the desired number of T_1_w images. An idle period is included between the acquisition of the last radial stack and the subsequent IR pulse to improve the SNR.^37^, ^38^

This acquisition scheme is used to acquire three TEs to enable water/fat separation.^39^, ^40^ The acquisition time of the k‐space center for each radial stack is referred to as the *effective TI* (TI_eff_).^37^ The T_1_w image with the longest TI_eff_ is acquired with a small flip angle to reduce T_1_ contrast and serve as the phase reference for the PS reconstruction.^28^

The projection angles of the radial stacks are selected based on a modified golden angle scheme, described by the following equation^37^, ^41^:

(1) $$\;\theta \left(p,e,i\right)=\left[N_{\mathrm{PR}}\cdot N_{\mathrm{TE}}\frac{k-1}{N_{\mathrm{TI}}}+N_{\mathrm{PR}}\cdot \frac{n-1}{N_{\mathrm{TI}}}+m-1\right]\cdot \frac{\pi }{\mathrm{GR}}.$$

Here, θ is the spoke angle of the radial stack p,i,e denote the indices of TI_eff_, IR‐block, and TE, respectively. $N_{\mathrm{PR}}$, $N_{\mathrm{TE}}$, and $N_{\mathrm{TI}}$ are the number of prescribed radial spokes, TE and TI_eff_. GR denotes the golden ratio (1.61803…). This sampling scheme produces unique and incoherent undersampling artifacts across all TEs and TI_eff_ to improve the performance of the image reconstruction.^35^, ^41^

### 2.3 Phase‐sensitive, model‐based, water/fat‐separated image reconstruction

The proposed method yields undersampled, volumetric T_1_w and chemical shift‐encoded data (Figure 2C). A 4D, subspace‐constrained, model‐based locally low rank (LLR) image reconstruction algorithm was developed in MatLab (MathWorks 2022b, Inc., Natick, MA) to reconstruct water/fat‐separated, variable T_1_w images from the raw k‐space jointly.^42^, ^43^ Prior to the proposed algorithm, preprocessing steps were carried out as described in earlier work.^37^ The proposed LLR reconstruction is formulated as the following linear optimization problem:

(2) $$\begin{array}{cc} \left[\begin{array}{l} {\hat{A}}_{w}\\ {\hat{A}}_{f} \end{array}\right] & =\underset{\left[\begin{array}{l} A_{w}\\ A_{f} \end{array}\right]}{\text{argmin}}\frac{1}{2}{\left\|W\cdot \left(S-F\cdot C\cdot U_{L}\cdot P\cdot \left[\begin{array}{l} A_{w}\\ A_{f} \end{array}\right]\right)\right\|}_{2}^{2}\\ & \;+\lambda \sum_{r}{\left\|R_{r}\left(\left[\begin{array}{l} A_{w}\\ A_{f} \end{array}\right]\right)\right\|}_{*} \end{array}$$

(3) $$\begin{array}{cc} P & =\left[\begin{array}{ccc} \exp\left\{i2\pi \psi TE_{1}\right\} & 0 & 0\\ 0 & \exp\left\{i2\pi \psi TE_{2}\right\} & 0\\ 0 & 0 & \exp\left\{i2\pi \psi TE_{3}\right\} \end{array}\right]\\ & \;\cdot \left[\begin{array}{cc} 1 & \sum_{p}r_{p}\exp\left\{i2\pi f_{p}TE_{1}\right\}\\ 1 & \sum_{p}r_{p}\exp\left\{i2\pi f_{p}TE_{2}\right\}\\ 1 & \sum_{p}r_{p}\exp\left\{i2\pi f_{p}TE_{3}\right\} \end{array}\right] \end{array}$$

(4) $$S=\left[s_{m,n}\right],A_{W,F}=\left[a_{l}\right],m\in \left[1,M\right],n\in \left[1,N\right],l\in \left[1,L\right].$$

Here, $A_{W,F}$ are 4D complex‐valued tensors (three spatial dimensions, and subspace coefficients) containing T_1_ subspace coefficients ($a_{l}$) of water‐ and fat‐separated images corresponding to the $l^{\mathrm{th}}$ basis function. ${\hat{A}}_{W,F}$ are the solutions that minimize the cost function in Equation 2. W is the respiratory weighting matrix, and S is a 6D tensor (three spatial dimensions, TE, TI_eff_, and coil index) containing T_1_w k‐space series ($s_{m,n}$) acquired at the $m^{\mathrm{th}}$ TI_eff_ and the $n^{\mathrm{th}}$ TE.^40^ The operators F, C, and $U_{L}$ are nonuniform fast Fourier transform,^44^ coil sensitivity, and subspace transforms, respectively. ψ is the B_0_ field inhomogeneity map in Hertz (Hz). The operator P includes the multi‐peak water/fat signal model,^45^, ^46^ with $f_{p}$ and $r_{p}$ are the frequency offsets in Hz and relative signal amplitudes of the multi (6)‐peak spectral model of fat.^47^, ^48^ λ is a unitless regularization factor.

The operator $R_{r}$ extracts a 3D patch, from each $a_{l}$, centered around pixel r. ${\left\|\cdot \right\|}_{*}$ is the nuclear norm. Prior to the reconstruction, (i) respiratory weights are derived from the recorded respiratory bellows signal^49^, ^50^; (ii) the ESPIRiT method is used to estimate coil sensitivities^51^; and (iii) ψ is estimated via a graph‐cut algorithm using low‐resolution multi‐echo images reconstructed using the nonuniform fast Fourier transform algorithm.^52^

The water/fat‐separated PSIR‐T_1_w images are reconstructed following the model‐based LLR reconstruction^28^, ^43^:

(5) $$I_{W}=U_{L}\cdot A_{W}$$

(6) $$I_{W,\text{PSIR}}=\text{real}\left(I_{W}\cdot \exp\left\{-i∠I_{W}\left(M\right)\right\}\right)$$

(7) $$I_{W}=\left[i_{m}\right],m\in \left[1,M\right],$$

where $I_{W}$ is a 4D tensor (three spatial dimensions, and TI_eff_) containing complex‐valued T_1_w water image series ($i_{m}$) at the $m^{\mathrm{th}}$ TI_eff_, and the subscript L denotes the number of dimensions of the subspace transform. M is the total number of T_1_w images, and the operator ∠ extracts the phase of the complex‐valued image. $I_{W,\text{PSIR}}$ is the real‐valued phase‐sensitive image series. Accordingly, in the following sections, we refer to the proposed method as *PSIR‐T*_*1*_ *w imaging*.

### 2.4 Water‐specific pseudo‐T_1_ mapping

The proposed image acquisition method builds upon the theoretical framework established by Look and Locker,^53^ where the longitudinal magnetization curve is sampled at multiple time points. Combined with an IR pulse, this acquisition scheme can be characterized as an IR–Look‐Locker MRI.^37^, ^54^, ^55^

The image reconstruction algorithm in section 2.3 yields water/fat‐separated variable T_1_w image series reconstructed at multiple TI_eff_. Subsequently, water‐only T_1_w images are used to estimate water‐specific T_1_ maps according to the following equations:

(8) $$\begin{array}{cc} S\left(n;T_{1}\right) & =\rho \left\{\left(1-E_{R}\right)\frac{1-c_{1}^{n-1}}{1-c_{1}}+\left[\left(1-E_{I}\right)\right.\right.\\ & \;\left.\left.-M_{1}E_{I}\right]\right\}c_{1}^{n-1}\sin\left(a_{1}\right),n\in \left[1,N_{1}\right] \end{array}$$

(9) $$S_{\mathrm{IR}-\mathrm{LL}}\left({\mathrm{TI}}_{\mathrm{eff}}\right)={\left.S\left(n;T_{1}\right)\right|}_{n=\frac{{\mathrm{TI}}_{\mathrm{eff}}-\mathrm{TI}}{\mathrm{TR}}+1},$$

where $S\left(n;T_{1}\right)$ describes the T_1_w signal equation for a single isochromat following the $n^{\mathrm{th}}$ RF pulse in an IR‐block. $S_{\mathrm{IR}-\mathrm{LL}}\left({\mathrm{TI}}_{\mathrm{eff}}\right)$ describes the signal equation for the T_1_w image series acquired with the proposed method. ρ is the complex proton density term of water. $a_{1}$ is the flip angle of readout RF pulses (Figure 2A). $N_{1}$ is the number of RF pulses with flip angle of $a_{1}$. The following equations describe the variables used in Equations 8 and 9 to shorten the expressions.

(10) $$\;t=-\cos\left(a_{2}\right)E_{I}E_{D},c_{1}=\cos\left(a_{1}\right)E_{R},c_{2}=\cos\left(a_{2}\right)E_{R}$$

(11) $$E_{I}=\exp\left(-\frac{\mathrm{TI}}{T_{1}}\right),E_{R}=\exp\left(-\frac{\mathrm{TR}}{T_{1}}\right),E_{D}=\exp\left(-\frac{\mathrm{TD}}{T_{1}}\right)$$

(12) $$\begin{array}{cc} M_{1} & =\frac{1}{1-tc_{1}^{N_{1}-1}c_{2}^{N_{2}}}\left\{\left(1-E_{D}\right)+\left[\left(1-E_{R}\right)\frac{1-c_{2}^{N_{2}}}{1-c_{2}}\right.\right.\\ & \;\left.\left.+\left(1-E_{R}\right)\frac{1-c_{1}^{N_{1}-1}}{1-c_{1}}c_{2}^{N_{2}}+\left(1-E_{I}\right){c_{1}^{N_{1}-1}c}_{2}^{N_{2}}\right]\cos\left(a_{2}\right)E_{D}\right\}, \end{array}$$

where TD denotes the idle time between the last TI_eff_ frame and the following IR‐pulse. $a_{2}$ and $N_{2}$ are the flip angle and the number of RF pulses used to acquire the phase reference. Because the water‐only T_1_w images are used in T_1_ estimation, these maps are corrected for the presence of fat. However, these maps are not corrected for B_1_ transmit power inhomogeneity and/or inversion efficiency. Accordingly, we will refer to these water‐specific maps as *pseudo‐T*_*1*_ maps.^37^, ^38^

## 3 METHODS

### 3.1 Phantom imaging study

A phantom containing multiple vials with different combinations of the water‐specific T_1_ and proton density fat fraction was constructed for validation of the proposed method. Peanut oil was used to modulate proton density fat fraction between 0% and 20% (00%), 100%), 20%) across three sets. In each set, the T_1_ was modulated between 200 and 1000 ms (200, 400, 600, 800, and 1000 ms) using varying concentrations of NiCl_2_ across the five vials.^56^ The vials were placed in a spherical housing bathed in a doped water solution to minimize *B*_0_ inhomogeneity.

Two sets of imaging experiments were conducted on a 3.0 T clinical MRI system (Signa Premier, GE Healthcare, Waukesha, WI) using a 48‐channel phased array head coil. The first set of experiments were conducted to demonstrate the performance of the model‐based image reconstruction algorithm (section 2.3). Two sets of images were acquired with 150 and 50 IR‐blocks to obtain 10‐fold and 30‐fold undersampling factors, respectively. The remaining imaging parameters were as described in Table 1 (PSIR‐T_1_w, 3.0 T). PSIR‐T_1_w images were reconstructed using: (i) the proposed algorithm (section 2.3), and (ii) a conventional LLR algorithm with a subsequent graph‐cut water/fat separation algorithm to serve as a reference to demonstrate the performance improvement of the proposed method.^52^

**TABLE 1:** Acquisition parameters for the patient imaging study.

	Nav‐T_1_w		PSIR‐T_1_w	
Imaging parameters	1.5 T	3.0 T	1.5 T	3.0 T
$\theta_{1}/\theta_{2}$ (Flip angles)	[30°, 35°]	[30°, 35°]	6°/3°	6°/3°
$N_{1}/N_{2}$	N/A	N/A	396/44	396/44
FOV (cm^3^)	[35, 50] × [21, 40] × [40]	[36, 40] × [29, 32] × [35, 40]	44 × 44 × 25.6	44 × 44 × 25.6
Matrix size	320 × [192, 256] × 64	320 × [256, 288] × 64	400 × 400 × 64	400 × 400 × 64
Bandwidth (kHz)	± 40	± [40–50]	± 125	± 125
TE_1_/∆ TE/TR (ms)	[2.21, 2.37]/[5.80, 6.02]	[1.84, 2.49]/[5.03, 6.98]	2/1.6/8.8	2.2/0.8/7.4
Echoes	1	1	3	3
TI_eff_/∆ TI_eff_ (ms)	N/A	N/A	138/397	120/334
TI_eff_ frames	N/A	N/A	10	10
TD (ms)	N/A	N/A	500	500
Partial Fourier in *k*_*z*_	[69%, 73%]	[69%, 73%]	69%	69%
Acceleration in *k*_*z*_	× 1	× 1.5	× 1	× 1
Acceleration in *k*_*y*_	× 1.5	× 1.5	× 1	× 1
IR‐blocks	N/A	N/A	120	150
Radial oversampling	N/A	N/A	× 1.5	× 1.5
Scan duration (min)	[1:13, 3:25]	[1:13, 3:16]	9:02	9:42

*Note*: Acquisition parameters for Nav‐T_1_w and PSIR‐T_1_w methods for the in vivo studies performed at 1.5 and 3.0 T. Imaging parameters for the Nav‐T_1_w images showed small variations across patients, due to adjustments in FOV, slice coverage, and so forth. These variations were reported in the table as a range ([min, max]).

Abbreviations: GA, gadoxetic acid; IR, inversion recovery; N/A, not applicable; Nav‐T_1_w, navigated T_1_‐weighted; PSIR‐T_1_w, phase‐sensitive inversion recovery T_1_‐weighted; TD, idle time; TI_eff_, effective TI.

The second set of experiments were conducted to demonstrate the method's capability to generate pseudo‐T_1_ maps. A reference T_1_ map was acquired using a confounder‐corrected T_1_ mapping method.^37^ The proposed method was acquired with the imaging parameters shown in Table 1 (PSIR‐T_1_w, 3.0 T). Image reconstruction parameters for both experiments (Equation 2) were λ = 0.0005 and 3D‐patch size = 5 × 5 × 5 mm^3^. An MR signal dictionary was generated via Bloch equation simulations based on the imaging parameters (Table 1), covering a range of T_1_ from 100 to 4000 ms. The temporal basis was then estimated via singular‐value decomposition of the generated signal dictionary.

### 3.2 In vivo imaging study

Patients were recruited after approval from the local institutional review board, and informed written consent was obtained in compliance with the Health Insurance Portability and Accountability Act. From September 2023 to August 2024, patients who were scheduled for a clinical contrast‐enhanced MRI exam for detection and characterization of FLLs, including metastases, were included in the study.

Imaging experiments were carried out on 1.5 T (Signa Artist, GE Healthcare, Waukesha, WI) and 3.0 T clinical MRI systems (Signa Architect/Premier, GE Healthcare). All imaging systems were equipped with an anterior array coil (AIR Coil, GE Healthcare) and embedded posterior coil elements in the table. Patients undergoing the local standard liver imaging protocol were administered 0.05 mmol/kg GA (Eovist, Bayer HealthCare Pharmaceuticals Inc., Whippany, NJ) intravenous bolus injection at a rate of 1.5 mL/s, followed by a 20 mL saline flush injected at the same rate.^21^, ^24^

Navigated T_1_w (Nav‐T_1_w) with fat suppression,^21^ which is our local standard of care T_1_w acquisition, was performed first, followed by the proposed PSIR‐T_1_w method. Both images were acquired during the HBP 15–20 min after the contrast administration. Nine distinct T_1_w images and a phase reference were acquired and reconstructed with the proposed method. The imaging parameters for the Nav‐T_1_w and PSIR‐T_1_w images are reported in Table 1.

PSIR‐T_1_w images were reconstructed offline using the proposed algorithm (section 2.3). Prior to reconstruction, PSIR‐T_1_w data were divided into four respiratory phases, and respiratory weights were chosen so that the PSIR‐T_1_w images were effectively reconstructed during end‐expiration.^37^, ^49^, ^50^ A total of four slices were discarded from the edges of the imaging volume due to variations in the slab profile for both PSIR‐T_1_w and Nav‐T_1_w imaging. Nav‐T_1_w images were reconstructed online using the installed commercial software (GE Healthcare).

### 3.3 Expert reader study

Two board‐certified radiologists (reader 1; reader 2), each with 7 years of experience in abdominal MRI, independently evaluated Nav‐T_1_w and PSIR‐T_1_w images. The reader study was completed in two stages: In the first stage, readers were tasked to indicate their preference in a side‐by‐side, pairwise comparison of randomly chosen 2D images using a forced‐choice comparison setup.^57^, ^58^ Here, nine PSIR‐T_1_w images, acquired and reconstructed with the proposed method, were compared among themselves to determine the PSIR‐T_1_w image with the optimal TI_eff_. Each pairwise comparison included two colocalized PSIR‐T_1_w 2D images with distinct TI_eff_ from the same patient. PSIR‐T_1_w images were ranked based on an Elo rating system.^59^

In the second stage, PSIR‐T_1_w images with the optimal TI_eff_ chosen in stage I were compared against the colocalized Nav‐T_1_w images from the same patient using ordinal 5‐point rating scales (5 = excellent, no artifacts; 4 = very good, minor artifacts; 3 = moderate quality and contrast, moderate artifacts; 2 = fair, major artifacts; 1 = poor, severe artifacts, nondiagnostic) to assess overall image quality, presence of artifacts that impair assessment of the liver, and liver–lesion contrast (LLC). Category‐wise comparison of pooled expert reader ratings was performed with Wilcoxon signed rank tests, assuming statistical significance for *p* values <0.05. Inter‐reader reliability was assessed by calculating Cohen's kappa. Interpretation of kappa statistics followed Landis and Koch (1.00–0.81 = almost perfect; 0.80–0.61 = substantial; 0.60–0.41 = moderate; 0.40–0.21 = fair; 0.20–0.00 slight; <0.00 poor agreement).^60^

Finally, signal intensity (SI) of the liver and lesions were measured by one radiologist for quantitative evaluation of LLC. LLCs were calculated based on the following equation^27^, ^61^:

(13) $$\mathrm{LLC}=\left|\frac{SI_{\text{lesion}}-SI_{\text{liver}}}{SI_{\text{liver}}}\right|.$$

Circular regions of interest (ROI) were used to measure tissue signal intensity and were placed manually within both the lesion and the adjacent liver parenchyma, avoiding major vessels, bile ducts, lesions, and periphery of the liver using Horos (v3.36, Horos Project, Nimble Co LLC, Annapolis, MD). Care was taken to avoid healthy liver parenchyma to measure lesion signal intensity. ROIs were propagated between the Nav‐T_1_w and PSIR‐T_1_w images using the copy and paste functionality. Small manual adjustments were made to the slice and in‐plane ROI placement to account for differences in breath‐holding positions between acquisitions and achieve optimal colocalization of signal measurements.

## 4 RESULTS

### 4.1 Phantom imaging study

**FIGURE 3:**
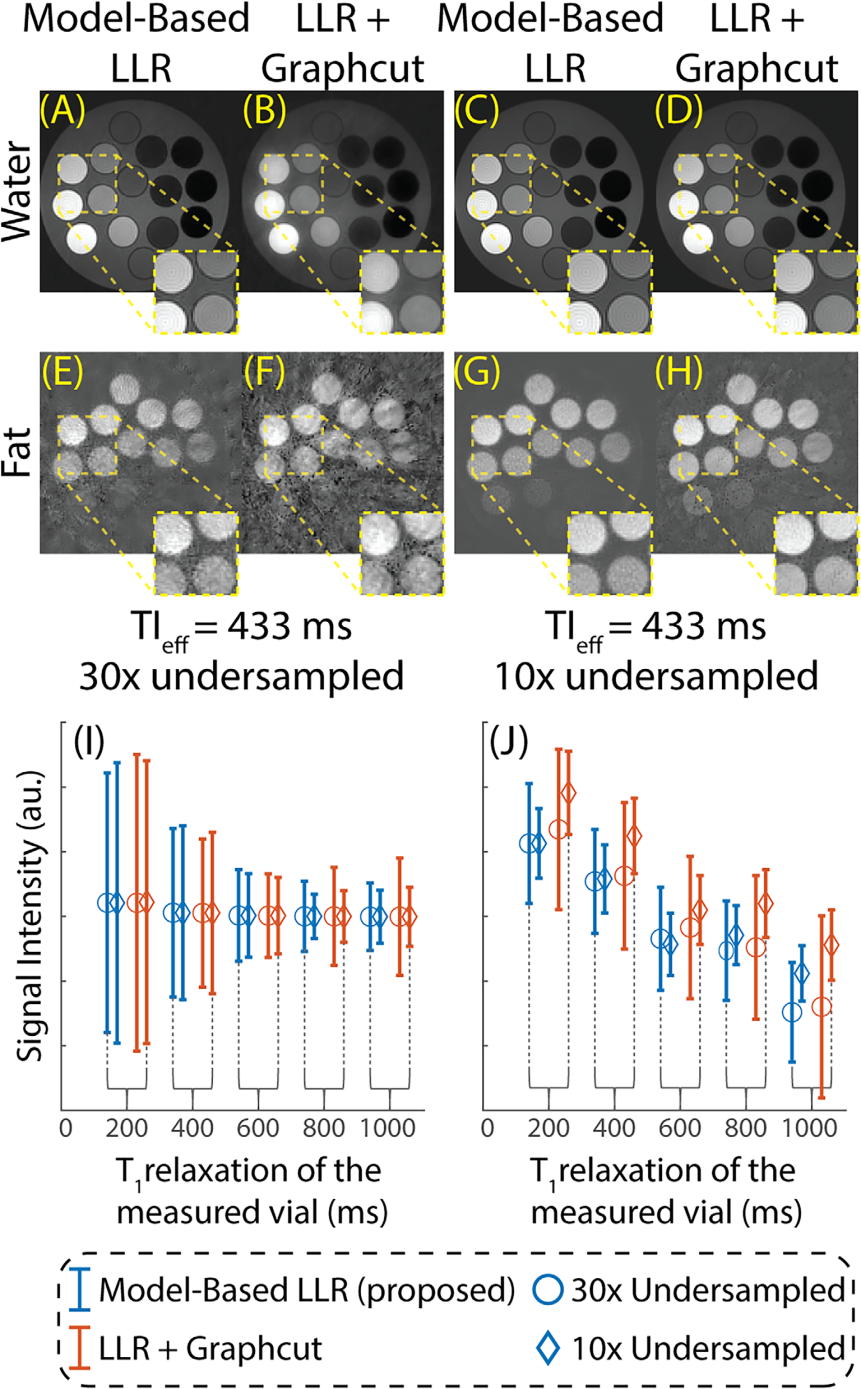
Water/fat‐separated PSIR‐T_1_w images are reconstructed using the proposed model‐based LLR method have better image quality and SNR performance than the LLR reconstruction with subsequent water/fat separation. (A, E, C, G) Images reconstructed using the proposed model‐based LLR method (section 2.3). (B, F, D, H) Images reconstructed using LLR method and a subsequent water/fat separation.^41^ The model‐based LLR method is robust against aggressive undersampling, as illustrated in (A, B, E, F). The superior SNR performance of the model‐based LLR is apparent in T_1_w fat images, even with less aggressive undersampling (E–H). (I, J) Normalized standard deviation is plotted, as a surrogate of SNR, for the water and fat signals for each reconstruction, respectively. Note that *x*‐axis of the plots represents the water‐specific T_1_ relaxation of the measured vial. LLR, locally low rank; PSIR‐T_1_w, phase‐sensitive inversion recovery T_1_‐weighted.

Figure 3 illustrates the performance of the proposed model‐based LLR method compared to a conventional LLR reconstruction with identical reconstruction parameters. Images are reconstructed at TI_eff_ of 433 ms. The model‐based LLR method (Figure 3A,C,E,G) achieved a robust image quality compared to conventional LLR reconstruction (Figure 3B,D–F) even with aggressive undersampling factors (e.g., 30‐fold). The normalized standard deviations are plotted against the water‐specific T_1_ relaxation of the corresponding vial as a surrogate for SNR performance (Figure 3I,J). The proposed method outperformed the conventional LLR reconstruction, yielding smaller standard deviations across different T_1_ vials, and water and fat images.

Figure S1 compares the reference confounder‐corrected T_1_ map (corrected for fat, $B_{1}^{+}$ inhomogeneity, and inversion efficiency) (Figure S1A) against the water/fat‐separated pseudo‐T_1_ maps reconstructed with the proposed method (Figure S1B).^37^ As noted in earlier work,^37^ the lack of $B_{1}^{+}$ inhomogeneity and inversion efficiency correction leads to some underestimation of T_1_ values. Accordingly, the method shows moderate linear agreement (slope = 0.83 (95% confidence interval [0.78 0.88]), intercept = 3 ms (95% confidence interval [−38 44]), r^2^ = 0.99) with confounder‐corrected T_1_ map (Figure S1C). Bland–Altman analysis revealed an average bias of −127 ± 70 ms (Figure S1D). The proposed method still mitigates the confounding effects of fat through water/fat separation as part of the acquisition and reconstruction (section 2.3).

### 4.2 In vivo imaging study

A total of 40 patients, 20 at 1.5 T (10 women, age = 50.8 ± 13.5 years, weight = 95.0 ± 30.2 kg) and 20 at 3.0 T (10 women, age = 51.2 ± 16.9 years, weight = 95.9 ± 21.2 kg), were successfully recruited and imaged. Lesion type and size distributions are presented in Table S1.

Figure S2 illustrates the image quality differences with respect to a subset of image reconstruction parameter pairs, with λ ranging from 0.0001 to 0.005, and 3D‐patch size ranging from 0.5 ×0.5×0. 5 mm^3^ to 30 × 30 × 30 mm^3^ at 3.0 T. Based on the empirical evidence, the image reconstruction parameters were chosen as: λ = 0.0005 (3.0 T), 0.001 (1.5 T) and 3D‐patch size = 5 × 5 × 5 mm^3^ (3.0 T), and 8 × 8 × 8 mm^3^ (1.5 T) for the remaining in vivo images.

**FIGURE 4:**
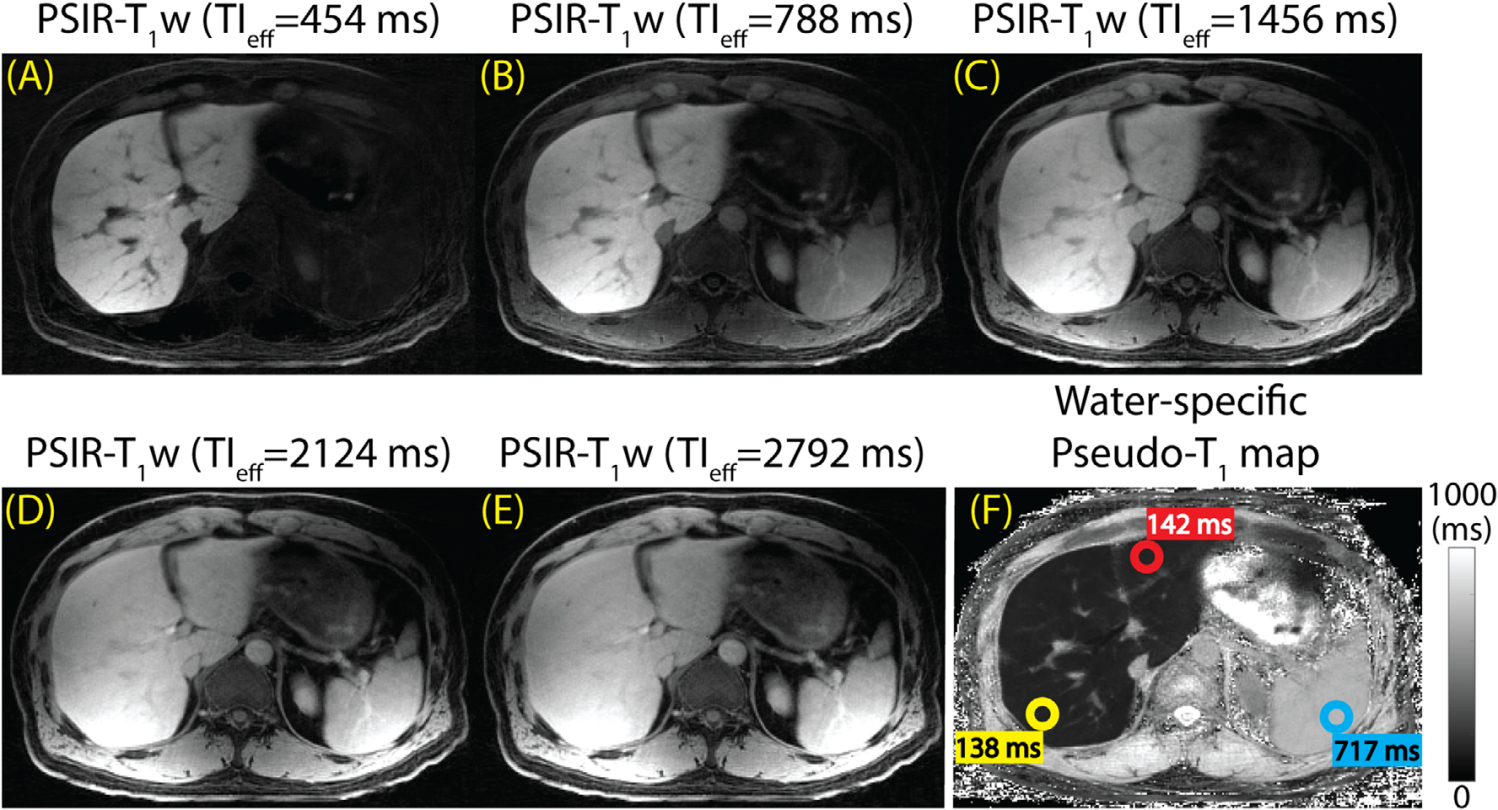
The proposed method allows probing of the T_1_ behavior of the tissues by producing multi‐contrast, PSIR‐T_1_w images of the liver. Multi‐contrast T_1_w images can be used to produce water‐specific pseudo‐T_1_ maps in a subsequent step. (A–E) PSIR‐T_1_w images acquired at TI_eff_ of 454, 788, 1456, 2124, and 2792 ms. (F) Pseudo‐T_1_ of liver was measured at 142 and 138 ms, in the left and right lobes, respectively. Pseudo‐T_1_ of spleen was measured at 717 ms.

Illustrated in Figure 4 are examples of the multi‐contrast PSIR‐T_1_w images and the pseudo‐T_1_ map acquired and reconstructed with the proposed method at 3.0 T. The method allows examination of T_1_ behaviors of different tissues (e.g., liver parenchyma, blood, muscle, spleen) across multiple TI_eff_. Figure 4A–E illustrates T_1_w images acquired at TI_eff_ of 454, 788, 1456, 2124, and 2792 ms, respectively. Figure 4F illustrates the pseudo‐T_1_ map with ROI measurements in the left liver lobe (red; 142 ms), right liver lobe (yellow; 138 ms), and spleen (blue; 717 ms). The model‐based LLR method reconstructs water/fat‐separated images directly, while suppressing streak artifacts and providing high image quality. Figure S3 displays all nine PSIR‐T_1_w images acquired from the same patient.

**FIGURE 5:**
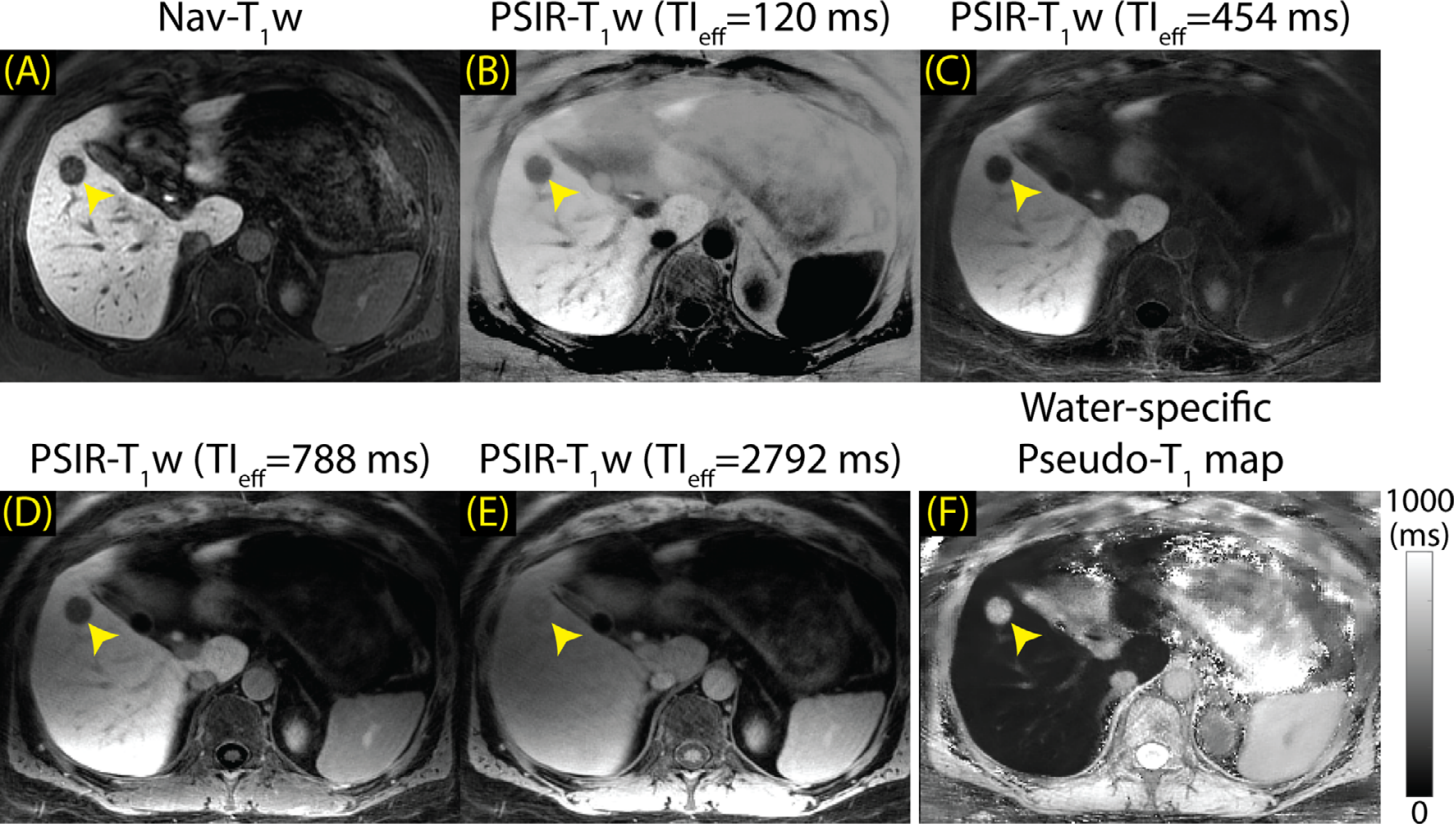
The proposed method improves lesion visibility and characterization by providing multi‐contrast PSIR‐T_1_w images in a single, free‐breathing scan (15 min post‐contrast). Liver metastasis is shown with a red arrowhead. (A) Nav‐T_1_w image has superior image quality. (B, C) PSIR‐T_1_w images acquired at TI_eff_ of 120 and 454 ms have higher LLC than the Nav‐T_1_w image despite the lower image quality. (D, E) PSIR‐T_1_w images acquired at TI_eff_ of 788 and 2792 ms exhibit lower LLC but good image quality. (F) Water‐specific pseudo‐T_1_ map reveals that the lesion has a longer pseudo‐T_1_ (687 ms) compared to liver parenchyma (141 ms). Nav‐T_1_w, navigated T_1_‐weighted.

Figure 5 compares the Nav‐T_1_w image (Figure 5A) to the corresponding PSIR‐T_1_w images (Figure 5B–E) and the pseudo‐T_1_ map (Figure 5F), acquired at 3.0 T in a patient with liver metastases. PSIR‐T_1_w images displayed in Figure 5B–E were acquired at TI_eff_ of 120, 454, 788, and 2792 ms. Although Nav‐T_1_w MRI had higher spatial resolution, PSIR‐T_1_w provides adjustable LLC. The PSIR‐T_1_w method provided good lesion visibility at TI_eff_ of 120, 454, and 788 ms (Figure 5B–D). Lesion visibility was poot at TI_eff_ of 2792 ms (Figure 5E). The lesion had a long pseudo‐T_1_ (687 ms) compared to liver parenchyma (141 ms) following the administration of GA. Blurring was observed in PSIR‐T_1_w images with shorter TI_eff_.

**FIGURE 6:**
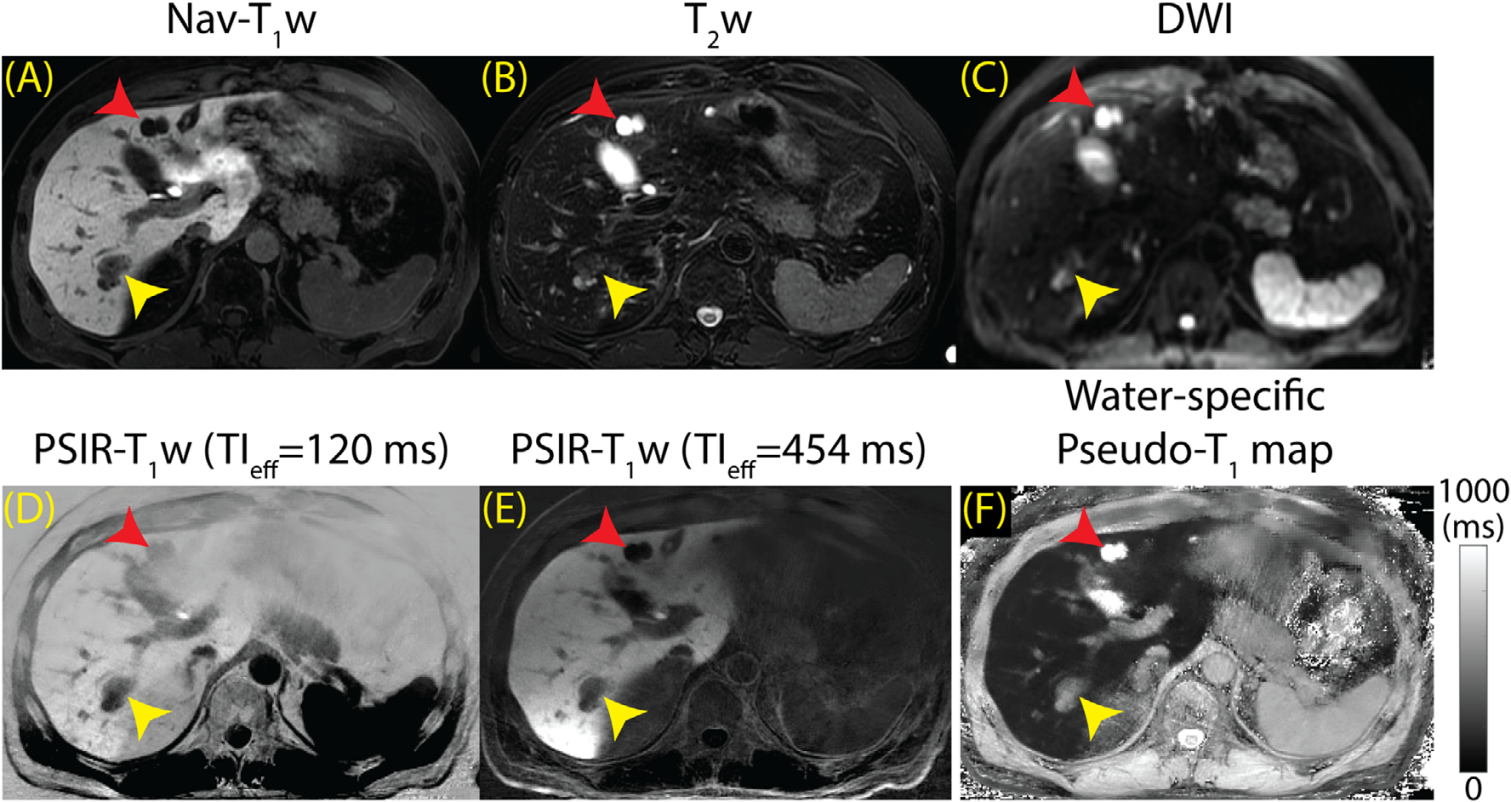
The proposed method improves lesion visibility and characterization by providing multi‐contrast PSIR‐T_1_w images in a single, free‐breathing scan (15 min post‐contrast). Liver metastasis and a simple cyst are shown with yellow and red arrowheads, respectively. (A) Nav‐T_1_w image has superior image quality compared with T_2_w and DWI MRI. (B, C) The simple cyst appears bright in T_2_w and DWI images, whereas metastasis is not visible. (D, E) Lesion visibility was good in PSIR‐T_1_w images acquired at TI_eff_ of 120 and 454 ms. (F) The simple cyst and metastasis had longer pseudo‐T_1_ of 1844 and 221 ms compared to 172 ms of liver parenchyma.

Figure 6 shows the Nav‐T_1_w (Figure 6A), T_2_w (Figure 6B), and DWI (Figure 6C) compared with the proposed PSIR‐T_1_w images (Figure 6D,E) and the pseudo‐T_1_ map (Figure 6F) at 3.0 T. PSIR‐T_1_w images displayed in Figure 6D,E were acquired at TI_eff_ of 120 and 454 ms. Nav‐T_1_w and T_2_w images had good image quality compared to DWI and the PSIR‐T_1_w method. The visibility of a simple cyst was good across all images (red arrowhead), whereas a metastasis was visible only in Nav‐T_1_w, PSIR‐T_1_w, and pseudo‐T_1_ map (yellow arrowhead). The simple cyst and metastasis had a longer pseudo‐T_1_ of 1844 ms and 459 ms compared to liver parenchyma (172 ms).

**FIGURE 7:**
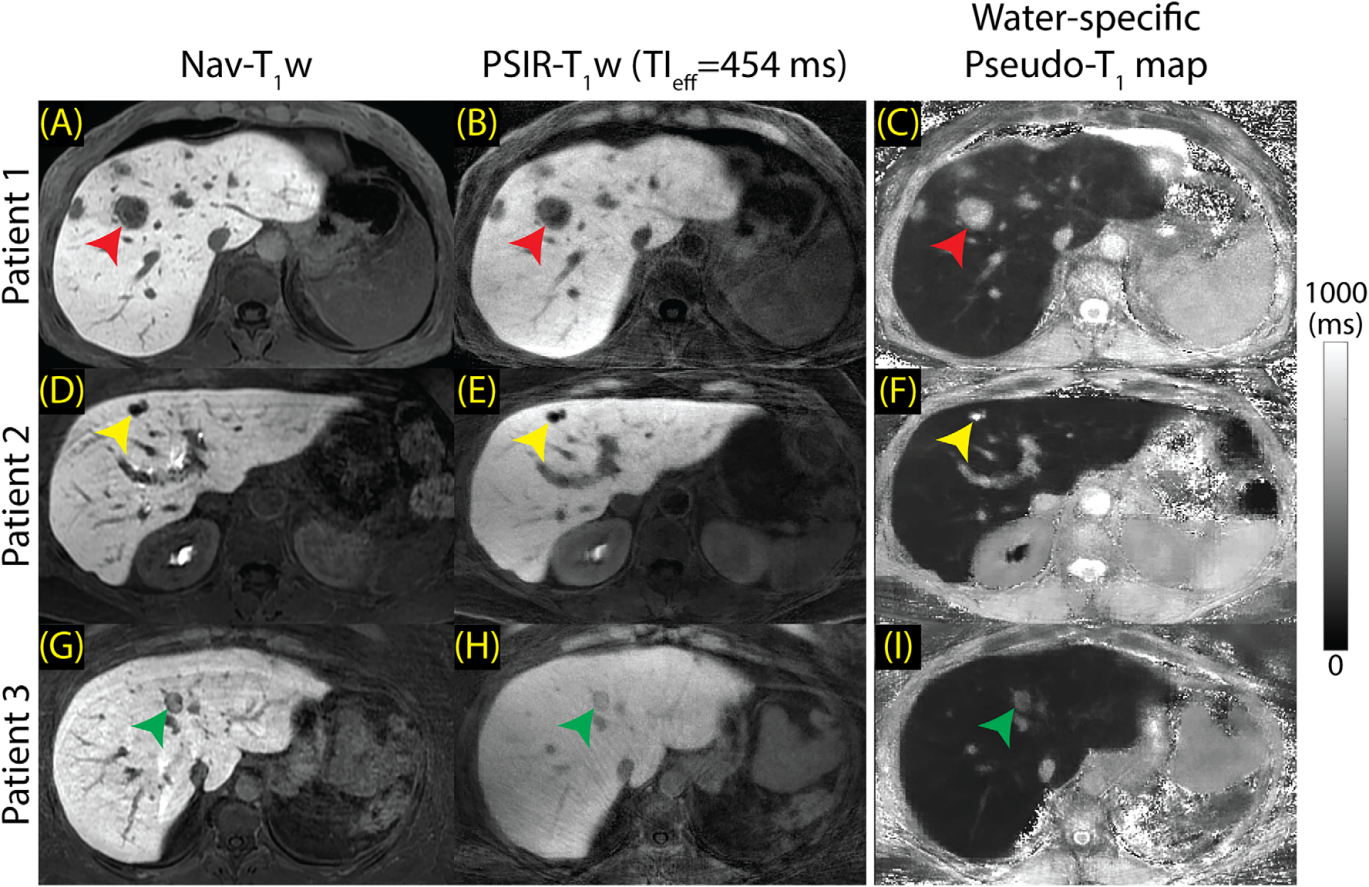
The proposed method improves lesion visibility and characterization on 1.5 T systems; however, it suffers from low SNR. Nav‐T_1_w and PSIR‐T_1_w images and pseudo‐T_1_ maps from three patients are presented (15 min post‐contrast). (A‐D) Red arrowhead points to a metastasis found in patient 1 with a pseudo‐T_1_ of 634 ms. (E‐H) Yellow arrowhead points to a simple cyst with pseudo‐T_1_ of 1216 ms. (I‐L) Green arrowhead points to an adenoma with a pseudo‐T_1_ of 321 ms.

Figure 7 shows comparisons of Nav‐T_1_w and PSIR‐T_1_w images and pseudo‐T_1_ maps acquired at 1.5 T in three separate patients. Shown are a metastasis (Figure 7A–C, red arrowhead), a simple cyst (Figure 7D–F, yellow arrowhead) and a hepatic adenoma (Figure 7G–I, green arrowhead) found in three patients. In patient 1, the T_1_ of metastasis was measured at 634 ms, compared to 208 ms of the liver; in patient 2, T_1_ of simple cyst was measured at 1216 ms, compared to 167 ms of the liver; and in patient 3, T_1_ of adenoma was measured at 321 ms, compared to 149 ms of the liver.

**FIGURE 8:**
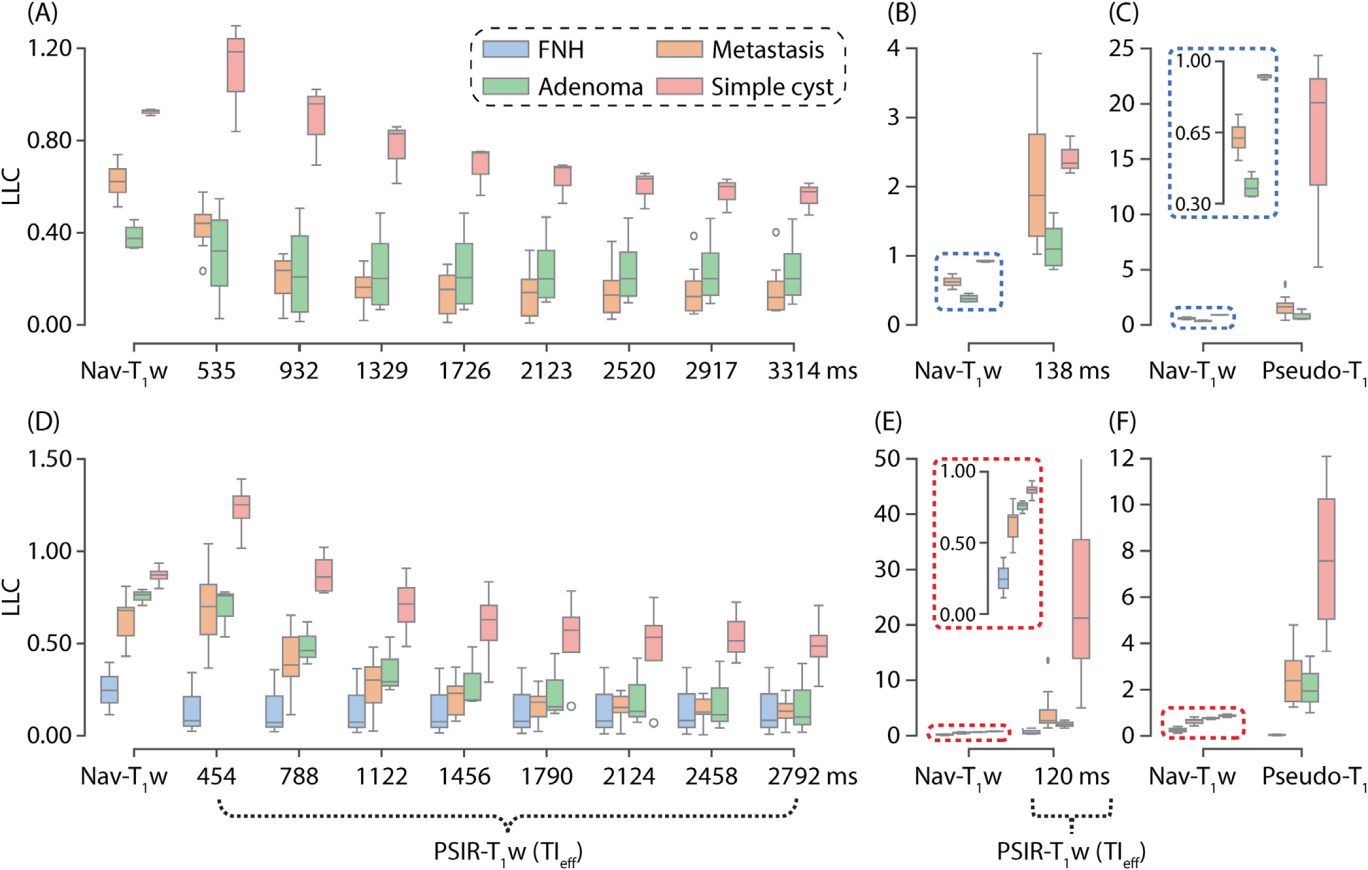
PSIR‐T_1_w images with shorter TI_eff_ and pseudo‐T_1_ maps had improved LLC compared to the clinical standard Nav‐T_1_w images, with the simple cyst having the highest across all images and both field strengths. PSIR‐T_1_w images acquired at the first and second TI_eff_ and the pseudo‐T_1_ map had wider LLC variations than the Nav‐T_1_w images. FNH was found in patients who were imaged at 3.0 T and had the lowest LLC. LLC reached a steady state in PSIR‐T_1_w images with longer TI_eff_ (>1400 ms). Metastasis and adenoma could not be distinguished based on their LLC. (A‐C) LLC comparisons at 1.5 T. (D‐F) LLC comparisons at 3.0 T. FNH, focal nodular hyperplasia; LLC, liver–lesion contrast.

Figure 8 contains LLC plots from all identified lesions, which included 16 lesions at 1.5 T and 46 lesions at 3.0 T, comparing Nav‐T_1_w and PSIR‐T_1_w imaging with varying TI_eff_. Figure 8A–C and Figure 8D–F display LLC comparison between the Nav‐T_1_w and the PSIR‐T_1_w at 1.5 T and 3.0 T, respectively. Figure 8A,D displays LLC comparisons between the Nav‐T_1_w and PSIR‐T_1_w images from the second to the ninth TI_eff_. Due to the large variations in LLC scale, LLC comparisons between the Nav‐T_1_w and the first TI_eff_ and pseudo‐T_1_ map are displayed separately in Figure 8B,C,E,F. PSIR‐T_1_w images acquired between the first and third TI_eff_, and pseudo‐T_1_ map consistently had higher LLC differences between simple cysts and metastases at both 1.5 T and 3.0 T. PSIR‐T_1_w images acquired at the first and second TI_eff_ (138 and 535 ms at 1.5 T, and 120 and 454 ms at 3.0 T), and the pseudo‐T_1_ map had wider LLC variations between different lesion types than the other images. After the third TI_eff_, PSIR‐T_1_w images had comparable LLC with the Nav‐T_1_w images; however, LLC in PSIR‐T_1_w images, both at 1.5 T and 3.0 T, had higher variation. LLC appears to reach a steady state beyond TI_eff_ of 1726 ms at 1.5 T, and 1456 ms at 3.0 T, where T_1_ contrast of the lesions changed minimally. In all images, the simple cyst had the highest LLC followed by metastasis, adenoma, and focal nodular hyperplasia (FNH). We note that, as expected, FNH, a benign hamartoma of hepatocyte origin, has similar signal intensity as liver, and therefore very low LLC, as shown in Figure 8F. Based on these data, PSIR‐T_1_w achieves superior LLC for cysts, compared to metastases and adenomas, with optimal TI_eff_ at approximately 535 ms and 454 ms at 1.5 T and 3.0 T, respectively.

Tables S2 and S3 show the numeric LLC differences including a statistical analysis calculating Mann Whitney U tests per sequence for LLC between cysts and metastases because this reflects the hypothesis of our study. Statistically, the contrast difference between cyst and metastasis was significant throughout all different TI_eff_; however, the ΔLLC was highest in the pseudo‐T_1_ map and in the first TI_eff_ (however, radiologists are not used to read inverted contrast images). Yet, the LLC difference was also particularly high in the second and third TI_eff_, which fits very well to the results of the expert reader study.

### 4.3 Expert reader study

Using the forced‐choice pairwise comparison tool, image pairs were randomly selected. Image rankings were stabilized after approximately 500 comparisons. To ensure robustness, comparisons were continued up to *N* = 1500 (reader 1/reader 2, 750 per reader) before concluding the first step of the expert reader study. This step was performed twice, separately for 1.5 and 3.0 T. PSIR‐T_1_w images acquired at TI_eff_ of 535 ms (at 1.5 T) and 454 ms (at 3.0 T) were deemed to provide the optimal LLC; hence, these data sets were compared with the Nav‐T_1_w images in the second step of the expert reader study.

In the scaled‐based image quality analysis, readers found no significant difference between the overall image quality of LLC‐optimized PSIR‐T_1_w and Nav‐T_1_w data sets (*p* = 0.46). Artifacts were less pronounced in the Nav‐T_1_w images (*p* < 0.001), with considerably more streak and blurring artifacts in PSIR‐T_1_w. Yet, observer‐based LLC was still deemed superior in PSIR‐T_1_w (*p* < 0.001). Detailed ratings are provided in Table 2. Inter‐reader reliability was substantial for overall image quality, artifacts, and subjective LLC, indicated by Cohen's kappa values of 0.77 (95% confidence interval 0.59–0.94, *p* < 0.001), 0.60 (0.47–0.76, *p* < 0.001), and 0.60 (0.40–0.79, *p* < 0.001), respectively.

**TABLE 2:** Image quality ratings determined by expert readers.

Rating (3.0 T)	Overall image quality		Artifact impairment		Subjective liver–lesion contrast	
	PSIR‐T_1_w	Nav‐T_1_w	PSIR‐T_1_w	Nav‐T_1_w	PSIR‐T_1_w	Nav‐T_1_w
5	8 (20.0%)	13 (32.5%)	0 (0%)	28 (70.0%)	18 (45.0%)	4 (10.0%)
4	29 (72.5%)	22 (55.0%)	11 (27.5%)	8 (20.0%)	18 (45.0%)	17 (42.5%)
3	1 (2.5%)	4 (10.0%)	14 (35.0%)	2 (5.0%)	4 (10.0%)	19 (47.5%)
2	2 (5.0%)	1 (2.5%)	12 (30.0%)	2 (5.0%)	0 (0%)	0 (0%)
1	0 (0%)	0 (0%)	3 (7.5%)	0 (0%)	0 (0%)	0 (0%)
Median (IQR)	4 (4–4)	4 (4–5)	3 (2–4)	5 (4–5)	4 (4–5)	4 (3–4)
**Rating (1.5 T)**						
5	0 (0%)	22 (55.0%)	0 (0%)	21 (52.5%)	7 (17.5%)	23 (57.5%)
4	19 (47.5%)	13 (32.5%)	12 (30.0%)	12 (20.0%)	16 (40.0%)	12 (30.0%)
3	16 (40.0%)	5 (12.5%)	16 (40.0%)	7 (17.5%)	13 (32.5%)	5 (12.5%)
2	5 (12.5%)	0 (0%)	12 (30.0%)	0 (0%)	4 (10%)	0 (0%)
1	0 (0%)	0 (0%)	0 (0%)	0 (0%)	0 (0%)	0 (0%)
Median (IQR)	3 (3–4)	5 (4–5)	3 (2–4)	5 (4–5)	4 (3–4)	5 (4–5)

*Note*: Pooled image quality ratings of two expert readers using an equidistant 5‐point scale (5 = excellent image quality and contrast, no artifacts; 4 = very good image quality and contrast, minor artifacts; 3 = moderate image quality and contrast, moderate artifacts; 2 = fair image quality and contrast, major artifacts; 1 = poor image quality and contrast, severe artifacts). Results for optimal PSIR‐T_1_w (TI_eff_ of 535 and 454 ms at 1.5 and 3.0 T, respectively) and Nav‐T_1_w are displayed as absolute and relative frequencies with median values and IQRs.

Abbreviations: IQR, interquartile range.

## 5 DISCUSSION

In this study, we proposed a novel free‐breathing, PSIR‐T_1_w imaging method for improved detection and characterization of liver lesions during HBP imaging with GA. This method combines magnetization preparation, water/fat separation, and SoS sampling methods to produce whole‐liver, water‐only T_1_w phase‐sensitive reconstructed images. This combination facilitates the availability of variable T_1_ contrast of lesions at multiple TI_eff_. Further, the method allows estimation of water‐specific pseudo‐T_1_ maps due to the acquisition of multiple T_1_w images from a single scan. The feasibility of the method was demonstrated through a patient study at both 1.5 T and 3.0 T. We also demonstrated significantly improved contrast between FLLs and liver parenchyma highlighting the potential of this strategy to improve the characterization of FLLs.

Currently, standard abdominal MRI protocols include GA‐enhanced T_1_w imaging for high‐resolution examinations of the liver to detect lesions, and IOP, T_2_w, DWI, and DCE‐MRI for lesion characterization. However, these sequences are generally unable to provide comparable spatial resolution because the GA‐enhanced T_1_w imaging performed in the HBP. Consequently, these imaging methods may miss small lesions (<1 cm), hindering the diagnostic performance of the abdominal MRI protocols.^21^, ^23^, ^24^, ^25^ The proposed PSIR‐T_1_w imaging has the potential to improve the detection and characterization of FLLs due to its high resolution, strong T_1_ contrast, and ability to probe T_1_ behavior of lesions at varying inversion times through variable T_1_w imaging and pseudo‐T_1_ mapping. Furthermore, free‐breathing, whole‐liver imaging provides robust image quality and high imaging efficiency compared to navigated image acquisition. Accordingly, the method has the potential to improve current multi‐sequence MRI protocols by reducing the number of indeterminate lesions, particularly in cancer patients being evaluated for hepatic resection, and ultimately aid surgical planning.

Most notably, our acquisition strategy enables differential LLC analysis of metastases and simple cysts, addressing a significant clinical challenge in characterizing small lesions that are often occult on T_2_w imaging and DWI. Whereas current multi‐sequence protocols adequately assess larger lesions, diagnostic challenges persist for smaller lesions in which conventional imaging may yield indeterminate findings. Rather than replacing comprehensive multi‐sequence imaging, this technique targets the specific clinical scenario in which enhanced spatial resolution and lesion contrast are critical for confident small lesion assessment, representing a methodological advancement that could benefit routine hepatobiliary phase T_1_w imaging.

In this work, we acquired and reconstructed nine separate T_1_w images (and one phase reference) with distinct TI_eff_ using the proposed PSIR‐T_1_w imaging method. This imaging parameter selection was made empirically. The PSIR‐T_1_w method, with images acquired at shorter TI_eff_ (<800 ms), produced higher differential LLC between lesion types than the Nav‐T_1_w images; however, shorter TI_eff_ images exhibited increased blurring compared to longer TI_eff_ images due to rapid changes in T_1_ contrast at these TI_eff_ times. Our results indicate that images with longer TI_eff_ (>1400 ms) added less diagnostic value because LLC remained approximately constant. Accordingly, the number of acquired TI_eff_ images can be reduced to shorten the scan time. However, we note that reducing the number of TI_eff_ images may hinder the method's intrinsic pseudo‐T_1_ mapping capability, which may have some utility for lesion characterization.

Our results indicate that PSIR‐T_1_w method can be used to identify simple cysts based on their high LLC and the corresponding high signal in T_1_ maps. As expected, FNH, which are hamartomas of hepatocyte origin, exhibited the lowest LLC in pseudo‐T_1_ maps and corresponding low LLC across all PSIR‐T_1_w. FNH is a benign, often asymptomatic and incidental lesion of hepatocytic hyperplasia that develops within the liver parenchyma.^62^, ^63^, ^64^ Our data are in line with the underlying physiology of this lesion because they take up GA similarly to normal liver. PSIR‐T_1_w was also able to discriminate metastatic lesions from cysts and FNH. Hepatocellular adenomas on the other hand have a heterogeneous appearance in HBP with no GA‐uptake (hepatocyte nuclear factor 1‐α or inflammatory) or variable GA‐update (β‐catenin positive). The hepatocyte nuclear factor 1‐α adenomas identified in this study were difficult to distinguish from metastases based on LLC alone. In practice, this does not represent a clinical dilemma because clinical history (e.g., female sex, age, use of oral contraceptives, history of colon cancer) are often adequate to differentiate these lesions. Further, both metastases and adenomas require biopsy and histological confirmation prior to subsequent management.

Our work, similar to past work in contrast‐enhanced MR angiography, MP‐GRASP, and XD‐GRASP, is part of the ongoing effort to develop effective free‐breathing image acquisition methods, utilizing SoS k‐space sampling.^31^, ^36^, ^41^, ^65^ Our method employs three‐echo IDEAL acquisition to optimize noise performance in water/fat separation, even in the presence of *B*_0_ inhomogeneities. Further, our approach utilizes a model‐based locally low‐rank reconstruction algorithm that incorporates water/fat separation directly into the image reconstruction process, in contrast to the spatial total variation‐based reconstruction used in MP‐GRASP. Most important, our work focuses specifically on post‐hepatobiliary phase T_1_w imaging for liver lesion detection and characterization, achieving higher spatial resolution at the expense of longer acquisition times, unlike MP‐GRASP, which is designed primarily for T_1_ mapping. Whereas XD‐GRASP also performs motion‐resolved dynamic contrast‐enhanced imaging for liver lesion assessment, it lacks variable T_1_ weighting capability, and its use of frequency‐selective fat suppression may be negatively impacted by B_0_ field inhomogeneities.

Our method has several limitations. In this work, we aimed to enhance the detection and characterization of the FLLs. Our findings suggest that PSIR‐T_1_w images provide a greater dynamic range for T_1_ contrast and high resolution, improving lesion visualization. Furthermore, we presented cases with various types of lesions (e.g., cysts and metastases) that can be characterized based on their T_1_ behavior across different TI_eff_ and their pseudo‐T_1_. However, a larger patient population with diverse disease backgrounds and a variety of FLLs are necessary to further optimize the method, validate our findings, and determine the clinical implications of the method. Similarly, benign cavernous hemangiomas are common lesions that are important to characterize and differentiate from metastases. Unfortunately, no hemangiomas were present in any of the 40 subjects enrolled in this study.

In this study, we consistently acquired Nav‐T_1_w images at the 15‐min mark, followed by PSIR‐T_1_w at around the 20‐min mark. Although it is possible that this consistent acquisition order may introduce systematic bias, the uptake of GA in the liver generally has plateaued by 15 min in most patients.^21^ Whereas our previous T_1_ mapping studies suggest relatively stable GA concentration in the liver between 15 and 20 min postcontrast, this finding may not directly translate to T_1_w signal characteristics, which may have different sensitivity to concentration changes than quantitative T_1_ measurements.^37^, ^56^ Future studies should consider randomizing acquisition order or implementing shorter intersequence delays to minimize any potential bias.

Due to the distinct visual characteristics of Cartesian images and radial SoS (e.g., the streak artifacts), it was not possible to blind readers to the imaging sequence type. However, readers were blinded to all other data. We acknowledge this as a study limitation that could potentially influence subjective assessments.

Due to the lack of $B_{1}^{+}$ and inversion efficiency correction, this method did not produce confounder‐corrected T_1_ relaxation measurements. Rather, pseudo‐T_1_ values were measured from the images acquired with different TI_eff_. Future work is necessary to assess the impact of confounders such as $B_{1}^{+}$ and inversion efficiency.

The model‐based image reconstruction was formulated as a linear, convex optimization problem to directly reconstruct water/fat‐separated images from the k‐space data. This was achieved by estimating the nonlinear *B*_0_ field inhomogeneity term in a prior step, using a graph‐cut algorithm.^52^ This approach was feasible due to the prior knowledge that *B*_0_ field inhomogeneity is a smoothly varying term in the absence of severe susceptibility variations.^66^, ^67^ The performance of the proposed linear optimization algorithm may be hindered if this condition is not satisfied. Conversely, nonlinear optimization is computationally more expensive and often has multiple local optima, resulting in sensitivity toward the initial conditions. Further work is necessary to investigate the performance of nonlinear optimization algorithms as well as the impact of image reconstruction parameters; however, this is beyond the scope of the current work.

The current implementation of the proposed method also suffered from long acquisition times. However, we did not employ parallel imaging along the slice dimension, which could shorten scan time significantly. Consequently, achieving high through‐plane resolution necessitates acquiring a large number of slices, prolonging the acquisition time for each TI_eff_ image. This leads to poor temporal resolution of TI_eff_ and severe blurring in shorter TI_eff_ image. One potential solution is to employ accelerated acquisition along the slice dimension.^68^ Such improvement in the image acquisition may also reduce blurring with shorter TI_eff_ frames, while improving both through‐plane and temporal resolution in the inversion time dimension. These future improvement opportunities may further enhance the diagnostic value of the proposed method.

The TI_eff_ values strictly depend on the slice prescription, such as the number of slices and the partial Fourier percentage, due to the sequential sampling in the *k*_*z*_ dimension. As a result, our method does not allow selection, and therefore optimization of independent TI_eff_ values. Future improvements can address this limitation through and randomized sampling of *k*_*z*_ encodes to provide greater flexibility in TI_eff_ selection.

## 6 CONCLUSION

Liver lesion characterization requires the acquisition of multiple MR images with different contrasts (e.g., T_1_w, T_2_w, DWI, DCE‐MRI, IOP T_1_w). These various MRI acquisition methods have varying spatial resolution and ability to visualize small lesions, making characterization of small lesions a challenging task. The proposed method improves T_1_ contrast through the use of IR preparation and phase sensitive reconstruction. Furthermore, the use of SoS k‐space sampling provides free‐breathing, volumetric coverage with inherent motion robustness. Initial validation and feasibility studies suggest that the proposed method improves lesion detection and characterization due to its strong T_1_ contrast and multi‐contrast imaging capabilities. Further parameter optimization, reduction in acquisition time, and further clinical evaluation are needed to demonstrate the potential of this promising strategy.

## CONFLICTS OF INTEREST

y.m., j.f.h., j.p.g., t.a.c., s.m., a.p., d.h., and s.b.r. do not have any relevant conflicts of interest to declare. Ty A. Cashen and Sagar Mandava are employees of GE Healthcare. Unrelated to this work, d.h. and s.b.r. have ownership interests in Calimetrix. Additionally, s.b.r., unrelated to this work, has ownership interests in Reveal Pharmaceuticals, Cellectar Biosciences, Elucent Medical, VistaAI, and RevOps, and provides consulting services to Protara Therapeutics. Further, the University of Wisconsin receives research support from GE Healthcare and Bracco Diagnostics, also unrelated to this work.

## Supporting information


**Figure S1.** Multi‐contrast, water/fat‐separated T_1_w images reconstructed with the proposed model‐based LLR method (Section 2.3) can be used to estimate water‐specific T_1_ values using Equation 9. (A, B) The confounding effects of fat are mitigated on both the confounder‐corrected and water/fat‐separated T_1_ maps through use of different correction methods. Water/fat‐separated T_1_ maps reconstructed following the model‐based LLR method generally underestimate the T_1_ values, due to the lack of $B_{1}^{+}$ inhomogeneity and inversion efficiency correction. (C, D) Regression and Bland–Altman analysis confirm the negative bias in water/fat‐separated T_1_ maps compared with the confounder‐corrected T_1_ mapping.
**Figure S2.** Image reconstruction parameters, for example, LLR regularization coefficient (*λ*) and 3D‐patch (block) size, were empirically chosen to maximize SNR without losing image details (e.g., anatomical structures and image contrast). A set of images were reconstructed using a range of regularization coefficients [1e‐4, 5e‐3], and block sizes [0.5, 30 mm^3^]. The following parameter sets outperformed the rest at 1.5 T and 3.0 T field strengths, respectively: (λ = 0.001, block size = 8 × 8 × 8 mm^3^), λ = 0.0005, block size = 5 × 5 × 5 mm^3^). Shown images are acquired with a 3.0 T system.
**Figure S3.** (A–I) All nine multi‐contrast, water/fat‐separated PSIR‐T_1_w images reconstructed with the proposed method at 3.0 T. (J) Water‐specific T_1_ map.
**Table S1.** Characteristics of analyzed lesions.
**Table S2.** PSIR T_1_w liver lesion contrast at 1.5 T is highest in pseudo‐T_1_ maps and reconstructions of 2nd and 3rd TI_eff_.
**Table S3.** PSIR T_1_w liver lesion contrast at 3.0 T is higher in pseudo‐T_1_ maps and all different TI_eff_ compared to Nav‐T_1_w.
