# Supplementary material for: Free‐breathing phase‐sensitive inversion recovery T1 ‐weighted imaging for improved visualization of focal liver lesions
Source: Magn Reson Med. 2025 Sep 8;95(1):314–31. doi: 10.1002/mrm.70042 (PMC12620167; doi:10.1002/mrm.70042)
Supplement: Supplementary file 1 — Figure S1. Multi‐contrast, water/fat‐separated T1w images reconstructed with the proposed model‐based LLR method (Section 2.3) can be used to estimate water‐specific T1 values using Equation 9. (A, B) The confounding effects of fat are mitigated on both the confounder‐corrected and water/fat‐separated T1 maps through use of different correction methods. Water/fat‐separated T1 maps reconstructed following the model‐based LLR method generally underestimate the T1 values, due to the lack of B1+ inhomogeneity and inversion efficiency correction. (C, D) Regression and Bland–Altman analysis confirm the negative bias in water/fat‐separated T1 maps compared with the confounder‐corrected T1 mapping. Figure S2. Image reconstruction parameters, for example, LLR regularization coefficient (λ) and 3D‐patch (block) size, were empirically chosen to maximize SNR without losing image details (e.g., anatomical structures and image contrast). A set of images were reconstructed using a range of regularization coefficients [1e‐4, 5e‐3], and block sizes [0.5, 30 mm3]. The following parameter sets outperformed the rest at 1.5 T and 3.0 T field strengths, respectively: (λ = 0.001, block size = 8 × 8 × 8 mm3), λ = 0.0005, block size = 5 × 5 × 5 mm3). Shown images are acquired with a 3.0 T system. Figure S3. (A–I) All nine multi‐contrast, water/fat‐separated PSIR‐T1w images reconstructed with the proposed method at 3.0 T. (J) Water‐specific T1 map. Table S1. Characteristics of analyzed lesions. Table S2. PSIR T1w liver lesion contrast at 1.5 T is highest in pseudo‐T1 maps and reconstructions of 2nd and 3rd TIeff. Table S3. PSIR T1w liver lesion contrast at 3.0 T is higher in pseudo‐T1 maps and all different TIeff compared to Nav‐T1w. [file MRM-95-314-s001.docx]

**Free-Breathing Phase-Sensitive Inversion Recovery T_1_ weighted Imaging for**

**Improved Visualization of Focal Liver Lesions**

Yavuz Muslu^1,2^, Julius Frederik Heidenreich^2^, Jan-Peter Grunz^2^,

Ty A Cashen^3^, Sagar Mandava^3^, Ali Pirasteh^2,4^,

Diego Hernando^1,2,4,5^, Scott B Reeder^1,2,4,6,7^

^1^Department of Biomedical Engineering, University of Wisconsin-Madison, Madison, WI, United States, ^2^Department of Radiology, University of Wisconsin-Madison, Madison, WI, United States, ^3^GE Healthcare, Waukesha, WI, United States, ^4^Department of Medical Physics, University of Wisconsin-Madison, ^5^Department of Electrical and Computer Engineering, University of Wisconsin-Madison, Madison, WI, United States, Madison, WI, United States, ^6^Department of Medicine, University of Wisconsin-Madison, Madison, WI, United States, ^7^Department of Emergency Medicine, University of Wisconsin-Madison, Madison, WI, United States

**Supporting Information**


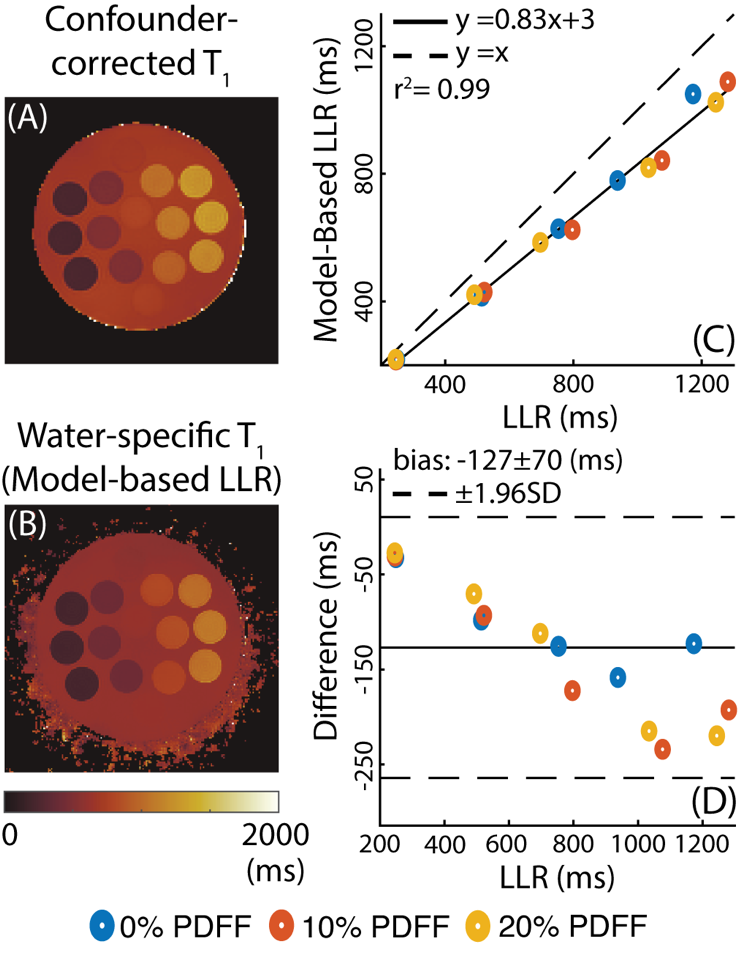


Figure S1: Multi-contrast, water/fat-separated T_1_w images reconstructed with the proposed model-based LLR method (Section 2.3) can be used to estimate water-specific T_1_ values using Equation 9. (A, B) The confounding effects of fat are mitigated on both the confounder-corrected and water/fat-separated T_1_ maps through use of different correction methods. Water/fat-separated T_1_ maps reconstructed following the model-based LLR method generally underestimate the T_1_ values, due to the lack of $B_{1}^{+}$ inhomogeneity and inversion efficiency correction. (C, D) Regression and Bland-Altman analysis confirm the negative bias in water/fat-separated T_1_ maps compared with the confounder-corrected T_1_ mapping.

**
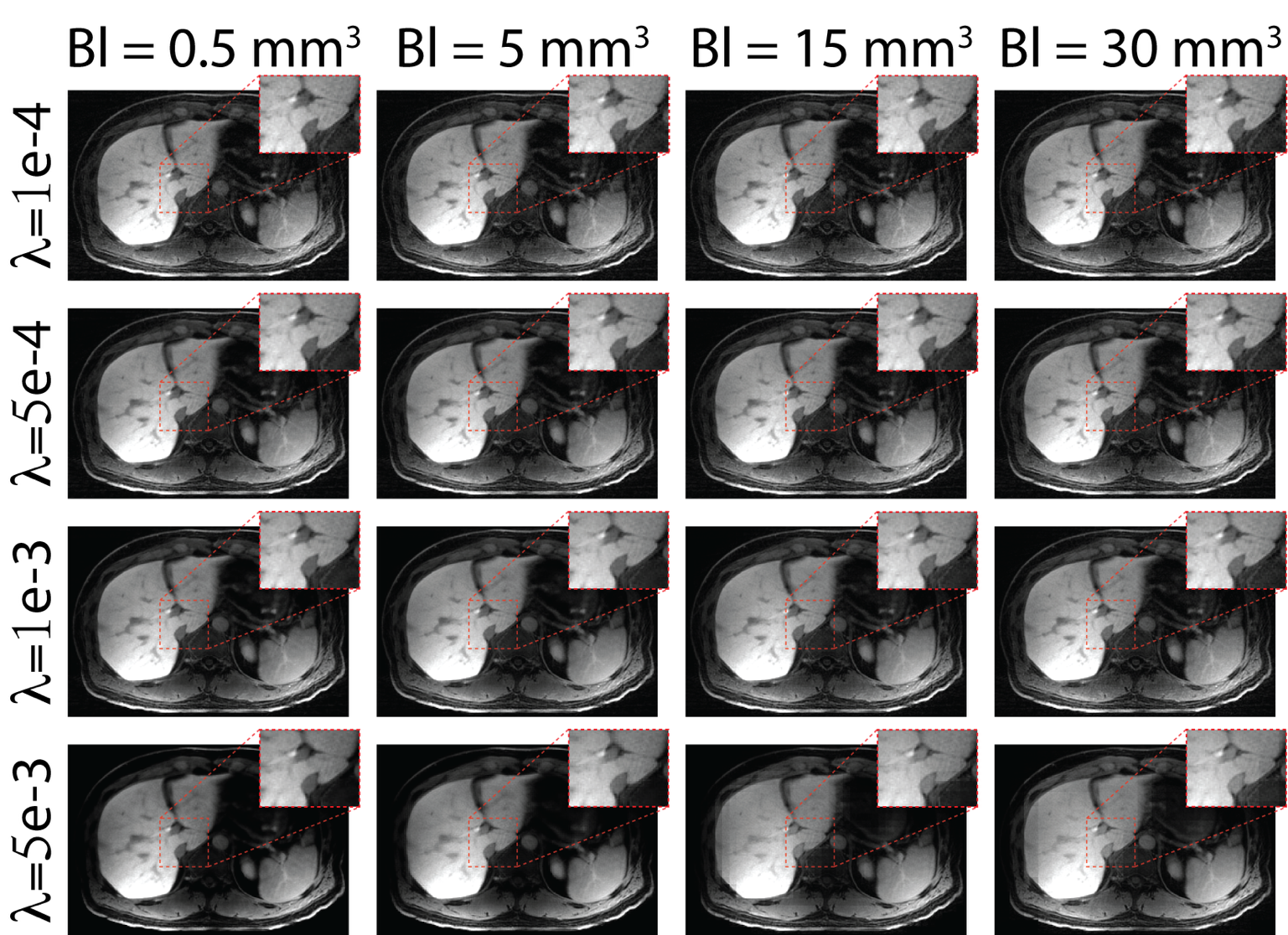
**

Figure S2: Image reconstruction parameters, e.g., LLR regularization coefficient (λ) and 3D-patch (block) size, were empirically chosen to maximize SNR without losing image details (e.g., anatomical structures and image contrast). A set of images were reconstructed using a range of regularization coefficients [1e-4, 5e-3], and block sizes [0.5 mm^3^, 30 mm^3^]. The following parameter sets outperformed the rest at 1.5 T and 3.0 T field strengths, respectively: ($\lambda$ = 0.001, block size = 8$\times$8$\times$8 mm^3^), $\lambda$ = 0.0005, block size = 5$\times$5$\times$5 mm^3^). Shown images are acquired with a 3.0 T system.


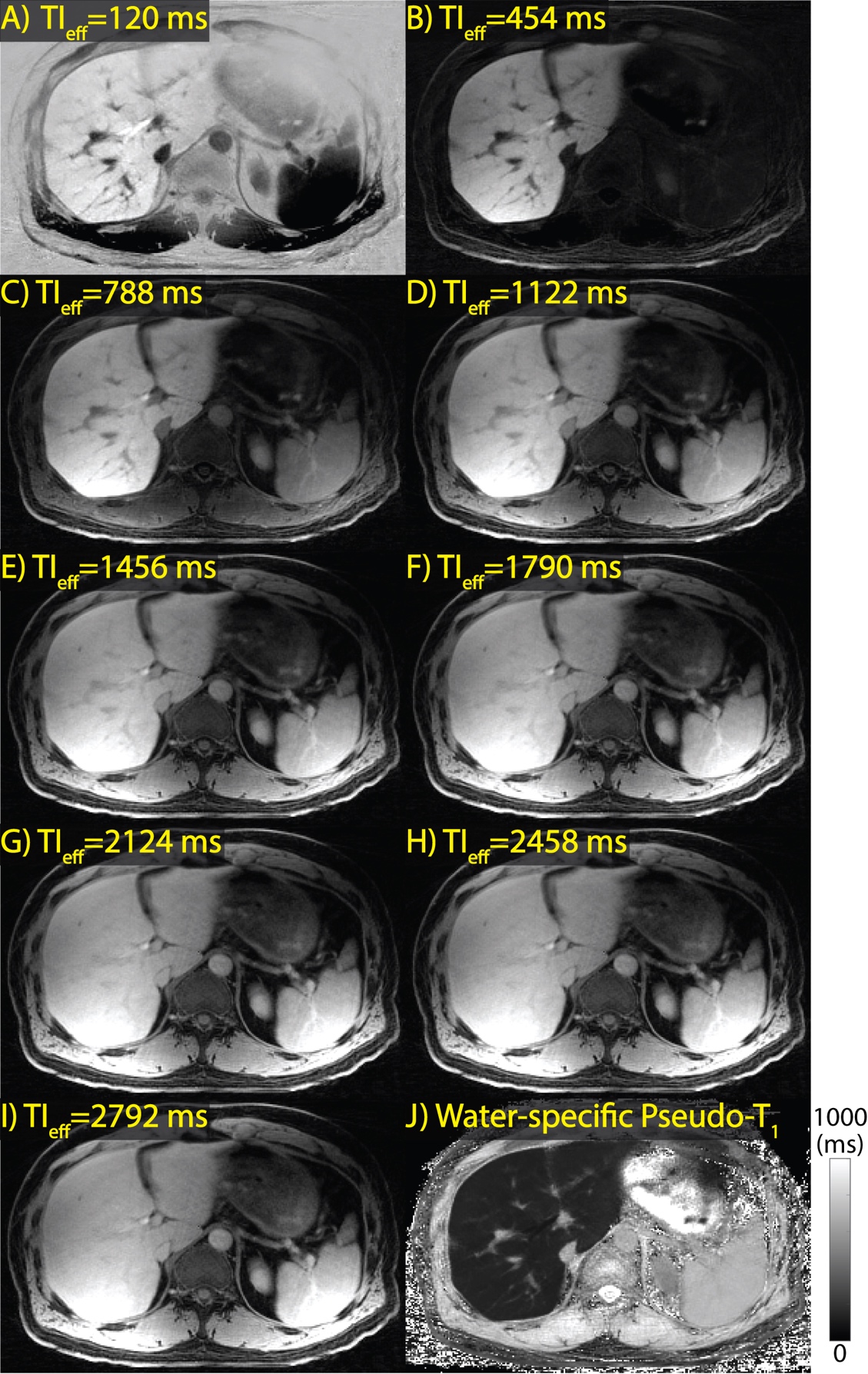


Figure S3: (A-I) All nine multi-contrast, water/fat-separated PSIR-T_1_w images reconstructed with the proposed method at 3.0 T. (J) Water-specific T_1_ map.

**Table S1**. Characteristics of analyzed lesions

| Lesion Type | Count (n) | Diameter (mm) |
| --- | --- | --- |
| 1.5 T | 16 |  |
| Cyst | 3 | 10 ± 6.7 (3.6 – 16.4) |
| Metastasis | 9 | 10 ± 3.3 (4.6 – 15.4) |
| Adenoma | 4 | 5.8 ± 0.2 (5.7 – 6.1) |
| 3.0 T | 46 |  |
| Cyst | 11 | 14.8 ± 12.8 (6.5 – 34.7) |
| Metastasis | 29 | 8.1 ± 2.6 (4.2 – 14.2) |
| Adenoma | 3 | 7.8 ± 2.2 (5.7 – 10.1) |
| FNH | 3 | 11.0 ± 1.9 (9.6 – 12.4) |

Note. – Diameter is shown in mean ± standard deviation and range in brackets

**Table S2.** PSIR T_1_w liver lesion contrast at 1.5T is highest in pseudo-T_1_ maps and reconstructions of 2^nd^ and 3^rd^ TI_eff_.

| **Sequence** | **LLC metastasis** | **LLC cyst** | **ΔLLC (cyst – metastasis)** | **P value** |
| --- | --- | --- | --- | --- |
| **Nav-T_1w_** | 0.64 (0.59 – 0.68) | 0.93 (0.92 – 0.93) | 0.29 | 0.004 |
| **PSIR-T_1w_** |  |  |  |  |
| **Pseudo-T_1_** | 1.37 (1.10 – 1.85) | 20.12 (12.68 – 22.25) | 18.75 | 0.004 |
| **TI_eff_ 1** | 2.19 (1.28 – 31.37) | 2.34 (2.27 – 2.53) | 0.14 | 0.84 |
| **TI_eff_ 2** | 0.44 (0.36 – 0.53) | 1.18 (1.01 – 1.24) | 0.74 | 0.004 |
| **TI_eff_ 3** | 0.22 (0.08 – 0.25) | 0.96 (0.83 – 0.99) | 0.74 | 0.009 |
| **TI_eff_ 4** | 0.17 (0.15 – 0.25) | 0.83 (0.72 – 0.84) | 0.66 | 0.004 |
| **TI_eff_ 5** | 0.19 (0.10 – 0.26) | 0.75 (0.65 – 0.75) | 0.56 | 0.004 |
| **TI_eff_ 6** | 0.18 (0.08 – 0.27) | 0.68 (0.60 – 0.69) | 0.50 | 0.004 |
| **TI_eff_ 7** | 0.18 (0.06 – 0.28) | 0.63 (0.57 – 0.65) | 0.46 | 0.004 |
| **TI_eff_ 8** | 0.18 (0.06 – 0.29) | 0.60 (0.54 – 0.62) | 0.43 | 0.004 |
| **TI_eff_ 9** | 0.16 (0.07 – 0.30) | 0.58 (0.53 – 0.60) | 0.41 | 0.004 |

Note - Data are shown as median with 25^th^ and 75^th^ quartile (brackets). LLC, liver lesion contrast. P value from Mann Whitney U test with level of significance at p < 0.05.

**Table S3.** PSIR T_1_w liver lesion contrast at 3.0T is higher in pseudo-T_1_ maps and all different TI_eff_ compared to Nav-T_1_w.

| **Sequence** | **LLC metastasis** | **LLC cyst** | **ΔLLC (cyst – metastasis)** | **P value** |
| --- | --- | --- | --- | --- |
| **Nav-T_1w_** | 0.68 (0.54 – 0.70) | 0.87 (0.85 – 0.89) | 0.19 | 0.003 |
| **PSIR-T_1w_** |  |  |  |  |
| **Pseudo-T_1_** | 2.37 (1.48 – 3.23) | 7.52 (5.01 – 10.18) | 5.15 | 0.006 |
| **TI_eff_ 1** | 2.89 (2.46 – 4.92) | 22.25 (14.64 – 37.02) | 19.36 | 0.006 |
| **TI_eff_ 2** | 0.68 (0.54 – 0.82) | 1.25 (1.18 – 1.30) | 0.55 | 0.003 |
| **TI_eff_ 3** | 0.38 (0.32 – 0.53) | 0.86 (0.79 – 0.95) | 0.48 | 0.001 |
| **TI_eff_ 4** | 0.30 (0.18 – 0.37) | 0.71 (0.62 – 0.80) | 0.41 | 0.001 |
| **TI_eff_ 5** | 0.23 (0.11 – 0.27) | 0.63 (0.52 – 0.71) | 0.40 | 0.006 |
| **TI_eff_ 6** | 0.18 (0.10 – 0.21) | 0.57 (0.45 – 0.64) | 0.39 | 0.05 |
| **TI_eff_ 7** | 0.15 (0.13 – 0.21) | 0.53 (0.41 – 0.60) | 0.38 | 0.10 |
| **TI_eff_ 8** | 0.13 (0.12 – 0.20) | 0.51 (0.47 – 0.57) | 0.38 | 0.001 |
| **TI_eff_ 9** | 0.13 (0.09 – 0.17) | 0.49 (0.43 – 0.54) | 0.35 | 0.001 |

Note - Data are shown as median with 25^th^ and 75^th^ quartile (brackets). LLC, liver lesion contrast. P value from Mann Whitney U test with level of significance at p < 0.05.
